# circPDE4B prevents articular cartilage degeneration and promotes repair by acting as a scaffold for RIC8A and MID1

**DOI:** 10.1136/annrheumdis-2021-219969

**Published:** 2021-05-26

**Authors:** Shuying Shen, Yute Yang, Panyang Shen, Jun Ma, Bin Fang, Qingxin Wang, Kefan Wang, Peihua Shi, Shunwu Fan, Xiangqian Fang

**Affiliations:** 1 Department of Orthopaedic Surgery, Sir Run Run Shaw Hospital, Zhejiang University school of medicine, Hangzhou, China; 2 Key Laboratory of Musculoskeletal System Degeneration and Regeneration Translational Research of Zhejiang Province, Hangzhou, China; 3 Department of Spine Surgery, The Central Hospital Affiliated to Shaoxing University, Shaoxing, China; 4 Department of Spine Surgery, The Hospital of the Marine Police Corps of the Chinese people's Armed Police Force, Jiaxing, China

**Keywords:** osteoarthritis, biological therapy, chondrocytes

## Abstract

**Objectives:**

Circular RNAs (circRNAs) have emerged as significant biological regulators. Herein, we aimed to elucidate the role of an unidentified circRNA (circPDE4B) that is reportedly downregulated in osteoarthritis (OA) tissues.

**Methods:**

The effects of circPDE4B were explored in human and mouse chondrocytes in vitro. Specifically, RNA pull-down (RPD)-mass spectrometry analysis (MS), immunoprecipitation, glutathione-S-transferase (GST) pull-down, RNA immunoprecipitation and RPD assays were performed to verify the interactions between circPDE4B and the RIC8 guanine nucleotide exchange factor A (RIC8A)/midline 1 (MID1) complex. A mouse model of OA was also employed to confirm the role of circPDE4B in OA pathogenesis in vivo.

**Results:**

circPDE4B regulates chondrocyte cell viability and extracellular matrix metabolism. Mechanistically, FUS RNA binding protein (FUS) was found to promote the splicing of circPDE4B, while downregulation of circPDE4B in OA is partially caused by upstream inhibition of FUS. Moreover, circPDE4B facilitates the association between RIC8A and MID1 by acting as a scaffold to promote RIC8A degradation through proteasomal degradation. Furthermore, ubiquitination of RIC8A at K415 abrogates RIC8A degradation. The circPDE4B–RIC8A axis was observed to play an important role in regulating downstream p38 mitogen-activated protein kinase (MAPK) signalling. Furthermore, delivery of a circPDE4B adeno-associated virus (AAV) abrogates the breakdown of cartilage matrix by medial meniscus destabilisation in mice, whereas a RIC8A AAV induces the opposite effect.

**Conclusion:**

This work highlights the function of the circPDE4B–RIC8A axis in OA joints, as well as its regulation of MAPK-p38, suggesting this axis as a potential therapeutic target for OA.

**Key messages**

**What is already known about this subject?**

- Circular RNAs broadly participate in normal physiology and disease, including functioning as miRNA sponges in osteoarthritis (OA).
- Protein post-translational modifications are necessary for proteins to perform physiological or pathological functions, including knee cartilage homeostasis.

**What does this study add?**

- circPDE4B serves as a scaffold to facilitate RIC8 guanine-nucleotide exchange factor A (RIC8A)–midline 1 binding, thereby decreasing RIC8A-dependent activation of the p38 mitogen-activated protein kinase signalling pathway and regulating OA progression.
- The role of RIC8A is first reported in chondrocytes, and K415 is found as the most important ubiquitination site of RIC8A regulated by circPDE4B.

**How might this impact on clinical practice or future developments?**

- The circPDE4B–RIC8A axis may serve as a potential therapeutic target for OA.

## Introduction

The aetiology of osteoarthritis (OA), the most common type of arthritis, is multifactorial and is associated with obesity, ageing, strain, trauma, congenital joint abnormalities and joint deformities.^1 2^ Although OA involves pathological changes in joint sites, including subchondral osteosclerosis, synovitis and osteophyte formation, destruction of cartilage represents its landmark.^3^ Considering that the extracellular matrix (ECM) accounts for 90% of the dry weight of cartilage,^4^ changes in its physiology directly impact the function of cartilage. Moreover, as the only cell type in cartilage, chondrocytes play an important role in maintaining ECM homeostasis.^5^ Thus, characterising the molecular mechanisms of chondrocytes involved in OA development and pathogenesis is crucial for improving prognosis and developing effective therapies.^6–8^

Recently, a growing number of studies have identified various functional non-coding RNAs, including circular RNAs (circRNAs), many of which are present in the human transcriptome.^9^ The expression of these circRNAs exhibits tissue specificity, while their heterocyclic structure makes them highly stable.^10^ Although circRNAs are believed to participate in cell differentiation and pluripotency,^11–13^ their specific functions remain largely uncharacterised. Moreover, although most identified circRNAs are non-coding, some have been recently described as protein coding.^14 15^ CircRNAs also have various biological functions related to different diseases.^16^ In fact, our previous report,^17^ as well as those of others,^18–20^ have reported a significant role for circRNAs in chondrocyte regulation of OA development and progression. However, these studies focused primarily on the function of circRNAs as miRNA sponges; hence, it remains unclear whether other molecular mechanism are also associated with the role of circRNAs in OA.

In the current study, we investigated the functions and molecular mechanisms of circPDE4B in OA. We believe that our study paves the way for future research investigating circRNA as a promising therapeutic target for OA.

## Methods

Detailed experimental procedures are described in the online supplemental materials and methods and tables.

10.1136/annrheumdis-2021-219969.supp1 Supplementary data

## Results

### circPDE4B exhibits lower expression in OA tissue

**Figure 1:**
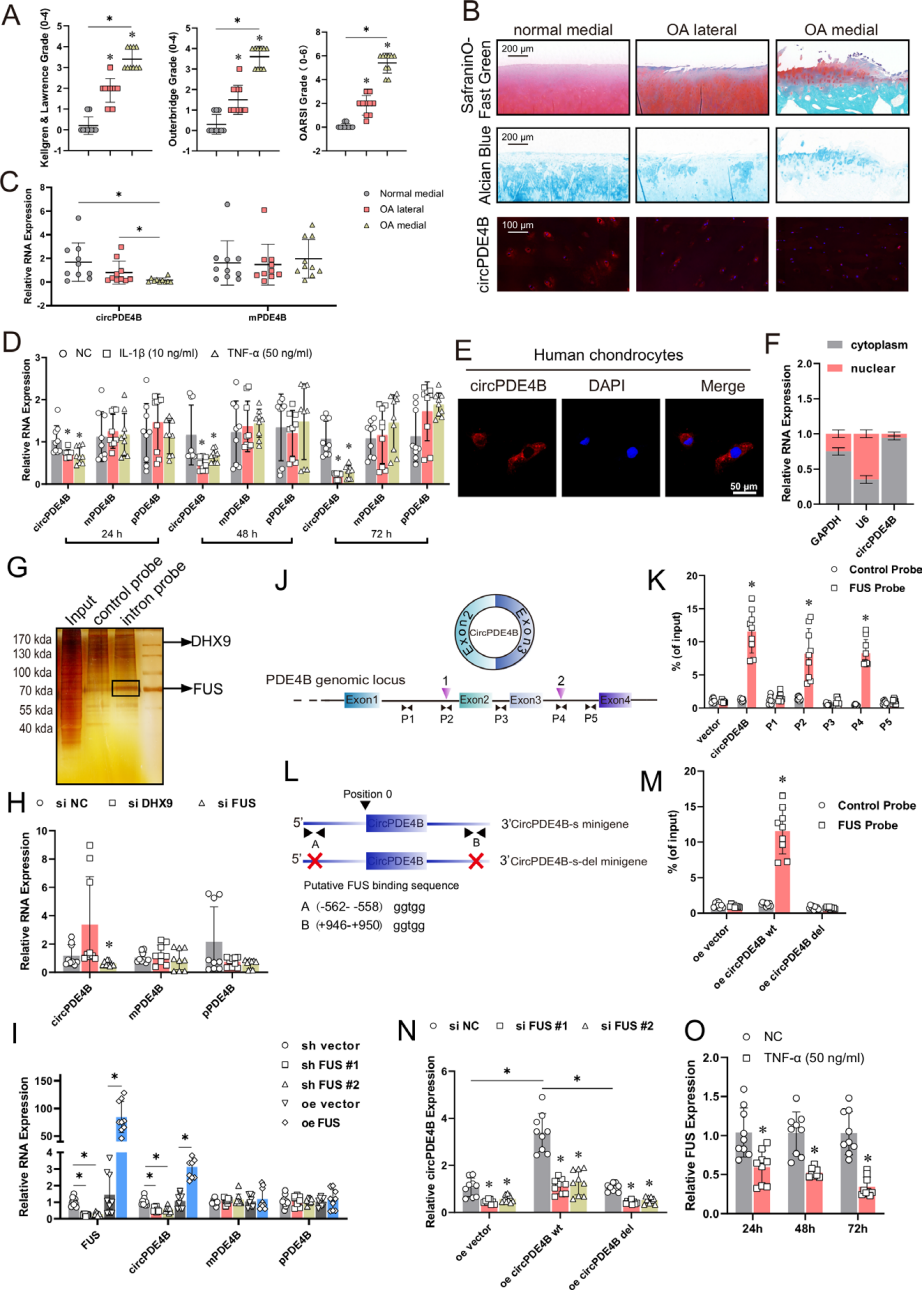
Characterisation of circPDE4B in human chondrocytes (HCs) and osteoarthritis tissues. (A) Preoperative Kellgren-Lawrence, Outerbridge and OARSI grading based for region of interest (ROI) cartilage (n=10 per group). *p≤0.05. (B) Histomorphological analysis and circPDE4B-labelled FISH staining for ROI cartilage. Scale bars, 200 μm. (C) circPDE4B and mPDE4B expression in ROI chondrocytes via RT-qPCR (n=10); *p≤0.05. (D) Changes in circPDE4B, mPDE4B and pPDE4B RNA levels, treated with IL-1β and TNF-α, assessed via RT-qPCR (n=9, 3 donors for three replicates); *p≤0.05. (E) Representative images of FISH staining for circPDE4B localisation in HCs. Scale bars, 50 µm. (F) Expression of circPDE4B assessed by RT-qPCR in the nuclear and cytoplasmic fractions (n=9, 3 donors for three replicates); *p≤0.05. (G) Silver staining of purified interaction proteins in the circPDE4B flanking sequence RPD experiment. (H) circPDE4B expression in HCs transfected with DExH-box helicase 9 and FUS siRNA or a negative control (n=9, 3 donors for three replicates); *p≤0.05. (I) circPDE4B, mPDE4B and pPDE4B expression after FUS inhibition or overexpression (n=9, 3 donors for three replicates); *p≤0.05. (J) Schematic of PDE4B pre-mRNA showing the locations of the two putative sites (inverted blue triangles) and amplicons (P1–P5) used for the RIP assay. (K) RIP assay performed with the PCR primers indicated in the schematic on the left. (n=9, 3 donors for three replicates); *p≤0.05. (L) Schematic illustrating the putative FUS-binding sites on the flanking introns in the circPDE4B-s minigene. The 5′ terminus of the circular exons of circPDE4B was defined as position 0. Putative FUS-binding sites A and B are located in the intron at the 5′ terminus of the circPDE4B exon (position: −562 to −558) and on the intron at the 3′ terminus of the circPDE4B exon (position: 946–950). (M) RIP analysis of FUS binding to circPDE4B-s and circPDE4B-s-del minigenes in HCs (n=9, 3 donors for three replicates); *p≤0.05. (N) Expression of circPDE4B relative to β-actin in HCs infected with circPDE4B-s or circPDE4B-s-del lentivirus followed by transfection with FUS siRNA or control siRNA (n=9, 3 donors for three replicates); *p≤0.05. (O) FUS mRNA expression level in HCs after TNF-α treatment (n=9, 3 donors for three replicates); *p≤0.05. DAPI, 4′,6-diamidino-2-phenylindole; FISH, fluorescence in situ hybridisation; FUS, FUS RNA binding protein; IL-1β, interleukin-1β; NC, negative control; RIP, RNA immunoprecipitation; RPD, RNA pull-down; RT-qPCR, quantitative reverse transcription PCR; TNF-α, tumour necrosis factor-α.

We previously performed RNA-seq analyses on the chondrocytes total RNA of ribosomal RNA deletion in three clinical OA and three control samples (SRA accession: PRJNA516555). Among the 50 most abundant significantly dysregulated circRNAs, the expression level of circPDE4B ranked first, the expression of which was significantly downregulated in chondrocytes of patients with OA (p<0.05, online supplemental table S1). In the current study, collected cartilage was assigned to one of three groups (total n=20): normal medial, OA lateral and OA medial. The OA severity for each case was assessed using the preoperative Kellgren-Lawrence, Outerbridge and Osteoarthritis Research Society International (OARSI) grading systems for the region of interest (ROI) (figure 1A). Meanwhile, histomorphological and western blot analyses accompanied by fluorescence in situ hybridisation (FISH) staining of ROI cartilage indicated that increased degradation of cartilage corresponded to decreased expression of circPDE4B in chondrocytes (figure 1B and online supplemental figure S1A). These results were confirmed by quantitative reverse transcription PCR (RT-qPCR) analysis which detected downregulated circPDE4B RNA levels in the chondrocytes of severe OA tissues, whereas mPDE4B mRNA level remained relatively consistent (figure 1C). Taken together, these results revealed that circPDE4B expression was negatively associated with OA severity.

10.1136/annrheumdis-2021-219969.supp2 Supplementary data

Considering that circPDE4B is conserved between humans and mice, we also detected circPDE4B expression in human/mouse chondrocytes (circPDE4B in human chondrocytes (HCs); circPde4b in mouse chondrocytes (MCs)) and found that interleukin-1β (IL-1β; 10 ng/mL) and tumor necrosis factor-α (TNF-α; 50 ng/mL) treatment significantly decreased circPDE4B/circPde4b expression in HCs/MCs in a time-dependent manner (figure 1D and online supplemental figure S1B). Moreover, Sanger sequencing displayed the splicing sequence of circPDE4B/circPde4b (online supplemental figure S1C, H). Meanwhile, circPDE4B/circPde4b was amplified by divergent primers from cDNA, but not in gDNA (online supplemental figure S1D, I). circPDE4B/circPde4b also exhibited a remarkable resistance to RNase R digestion (online supplemental figure S1E, J) and actinomycin D treatment (online supplemental figure S1F, K). Besides, mPDE4B/mPde4b was amplified by random primer and oligo(dT) primer, whereas circPDE4B/circPde4b was only amplified using random primers (online supplemental figure S1G, L). Nuclear separation experiments coupled with RT-qPCR analysis and FISH revealed that circPDE4B/circPde4b is primarily located in the cytoplasm of HCs/MCs (figure 1E, F and online supplemental figure S1M, N). Cumulatively, these results indicate that circPDE4B is downregulated in OA and, thus, may contribute to OA progression.

### FUS RNA binding protein (FUS) regulates circPDE4B expression through direct binding to pre-mRNA

We next sought to identify circPDE4B upstream regulators. We first performed RNA pull-down (RPD)-MS assay of circPDE4B flanking sequence and found two RNA splicing related RBPs, including DExH-box helicase 9 and FUS (figure 1G). RT-qPCR results indicated that following FUS knockdown, circPDE4B was downregulated in HCs, while pPDE4B and mPDE4B did not exhibit significant changes (figure 1H and online supplemental figure S2A). In addition, infection with two FUS shRNA lentivirus served to only decrease the expression of circPDE4B (figure 1I and online supplemental figure S2B), whereas overexpressed FUS upregulated the expression of circPDE4B (figure 1I). Next, RNA immunoprecipitation (RIP) assays revealed that FUS binds to exon-adjacent sites, while remote regions elsewhere were negligible (figure 1J, K). We also searched for potential FUS response elements and found two potential motifs, A located upstream and B located downstream. We further engineered two short circPDE4B minigenes, including circPDE4B-s and circPDE4B-s-del (figure 1L). RIP revealed an overt interaction between FUS and circPDE4B-s, but not with circPDE4B-s-del (figure 1M), indicating that FUS requires the putative sites in surrounding introns for binding. We next knocked down FUS in circPDE4B-s/del expressed HCs and found that circPDE4B-s had significantly reduced circPDE4B transcripts on FUS knockdown, compared with circPDE4B-del (figure 1N). Notably, FUS was downregulated by TNF-α in HCs (figure 1O). Cumulatively, the downregulation of circPDE4B in OA was, at least in part, caused by the inhibition of FUS.

10.1136/annrheumdis-2021-219969.supp3 Supplementary data

### circPDE4B regulates chondrocyte cell viability and ECM metabolism

**Figure 2:**
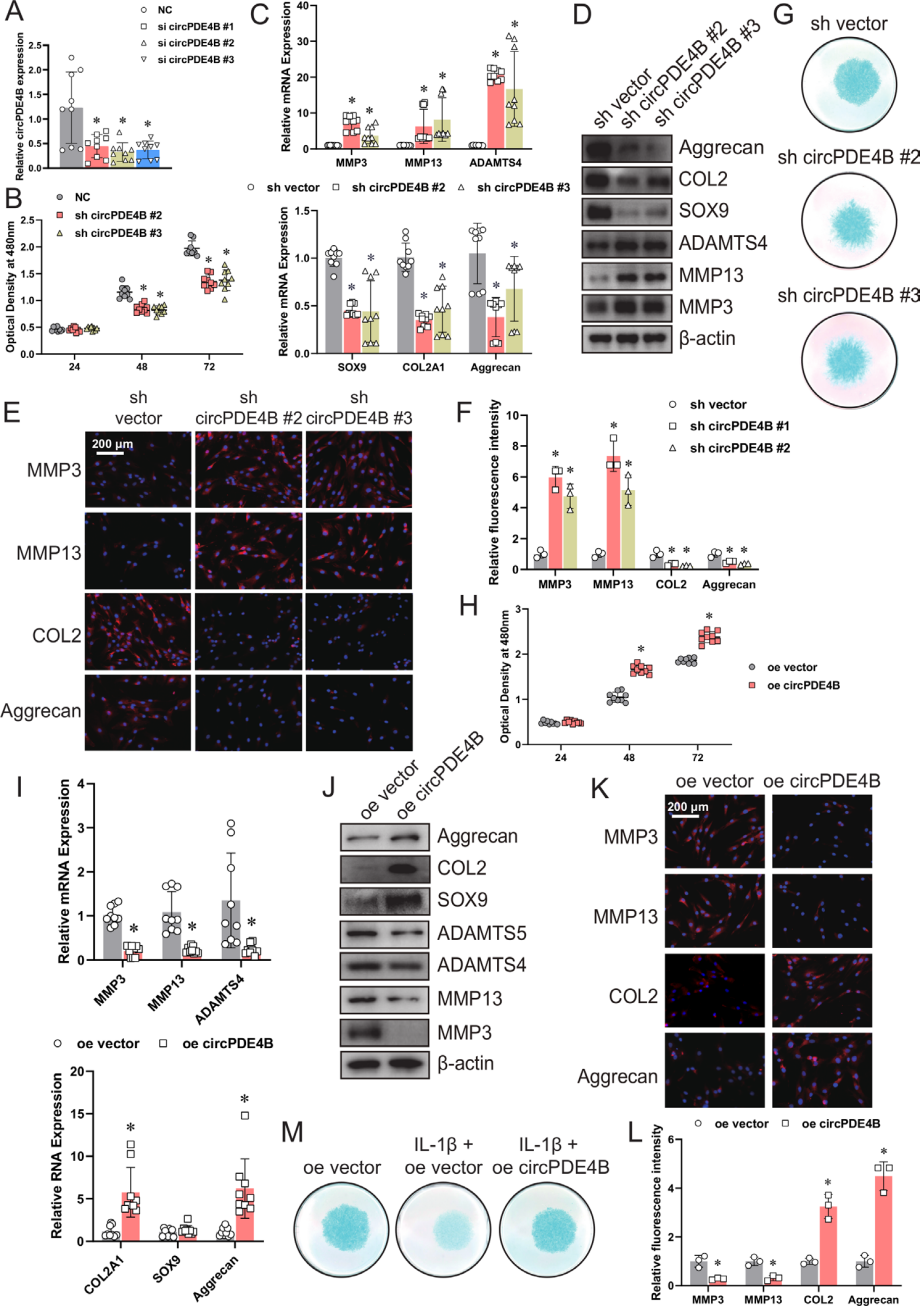
Targeting circPDE4B expression affects matrix-degrading and anabolic factors in human chondrocytes (HCs). (A) circPDE4B expression in HCs transfected with circPDE4B siRNAs or negative control siRNA (n=9, 3 donors for three replicates); *p≤0.05. (B) Viability of HCs infected with circPDE4B shRNA #2/#3 adenovirus or control shRNA adenovirus (n=9, 3 donors for three replicates); *p≤0.05. (C, D) mRNA and protein levels of MMP3, MMP13, ADAMTS4, COL2A1 (or COL2 protein), SOX9 and aggrecan in HCs infected with circPDE4B shRNA #2/#3 adenovirus or control shRNA adenovirus (n=9, 3 donors for three replicates); *p≤0.05. (E) Immunofluorescence analysis of MMP3, MMP13, COL2 and aggrecan. (F) Quantification of immunofluorescence analysis (n=9, 3 donors for three replicates); *p≤0.05. (G) Alcian blue staining of shRNA-treated HCs. (H) Viability of HCs infected with a circPDE4B overexpression adenovirus or control adenovirus (n=9, 3 donors for three replicates); *p≤0.05. (I, J) mRNA and protein levels of MMP3, MMP13, ADAMTS4, COL2A1 (or COL2 protein), SOX9 and aggrecan (n=9, 3 donors for three replicates); *p≤0.05. (K) Immunofluorescence of MMP3, MMP13, COL2 and aggrecan. (L) Quantification of immunofluorescence analysis (n=9, 3 donors for three replicates); *p≤0.05. (M) Alcian blue staining of HCs treated with IL-1β with or without circPDE4B overexpression. IL-1β, interleukin-1β; NC, negative control.

To assess the involvement of circPDE4B/circPde4b in ECM metabolism, we transfected HCs/MCs with three circPDE4B/circPde4b siRNAs, respectively (figure 2A and online supplemental figure S3A). Knockdown of circPDE4B/circPde4b expression did not affect PDE4B/Pde4b mRNA levels (online supplemental figure S3B, C).

10.1136/annrheumdis-2021-219969.supp4 Supplementary data

We then assessed the influence of circPDE4B/circPde4b on chondrocytes viability using a cell counting kit-8 (CCK-8) assay. Results showed that knockdown of circPDE4B/circPde4b expression reduced chondrocytes viability (figure 2B and online supplemental figure S3D). In addition, the inhibition of circPDE4B/circPde4b by shRNA adenovirus (online supplemental figure S3E, F) significantly enhanced the expression of MMP3, MMP13 and ADAMTS4, whereas the expression of SOX9, COL2A1 (or COL2 protein) and aggrecan was downregulated in HCs/MCs, as revealed by RT-qPCR (figure 2C and online supplemental figure S3G) and western blot (figure 2D and online supplemental figure S3H). Immunofluorescence further confirmed that circPDE4B/circPde4b knockdown affected MMP3, MMP13, COL2 and aggrecan levels in HCs/MCs (figure 2E, F and online supplemental figure S3I, J). Meanwhile, Alcian blue staining of HCs/MCs revealed that circPDE4B/circPde4b inhibition led to a chondrocytes dysfunction with less blue-stained proteoglycan. (figure 2G and online supplemental figure S3K).

We then performed gain-of-function experiments (online supplemental figure S3L, M) and found that overexpression of circPDE4B/circPde4b increased the viability of chondrocyte cells, as revealed by a CCK-8 assay (figure 2H and online supplemental figure S3N). Besides, mRNA and protein levels of MMP3, MMP13 and ADAMTS4 were downregulated, whereas those of SOX9, COL2A1 (or COL2 protein) and aggrecan were upregulated in circPDE4B/circPde4b-overexpressing HCs/MCs (figure 2I–L and online supplemental figure S3O–R). Furthermore, Alcian blue staining of HCs/MCs indicated that circPDE4B/circPde4b overexpression and IL-1β cotreatment reduced cartilage destruction compared with IL-1β treatment alone (figure 2M and online supplemental figure S3S). These data demonstrate that circPDE4B/circPde4b in HCs/MCs can promote cell viability and inhibit the catabolic effect.

### RIC8 guanine-nucleotide exchange factor A (RIC8A) interacts with circPDE4B and participates in OA

Cytoplasm-localised circRNAs participate in translational regulation by acting as ceRNAs, coding RNAs or as a scaffold for RBPs. AGO2 RIP assay revealed that circPDE4B does not bind to AGO2 (online supplemental figure S4A). Bioinformatics analysis of circPDE4B further revealed that it has an open reading frame (ORF) fragment (online supplemental figure S4B). Therefore, two full-length (FL) predicted ORFs were cloned into a eukaryotic expression vector, however, circPDE4B was not found to encode a protein (online supplemental figure S4C).

10.1136/annrheumdis-2021-219969.supp5 Supplementary data

**Figure 3:**
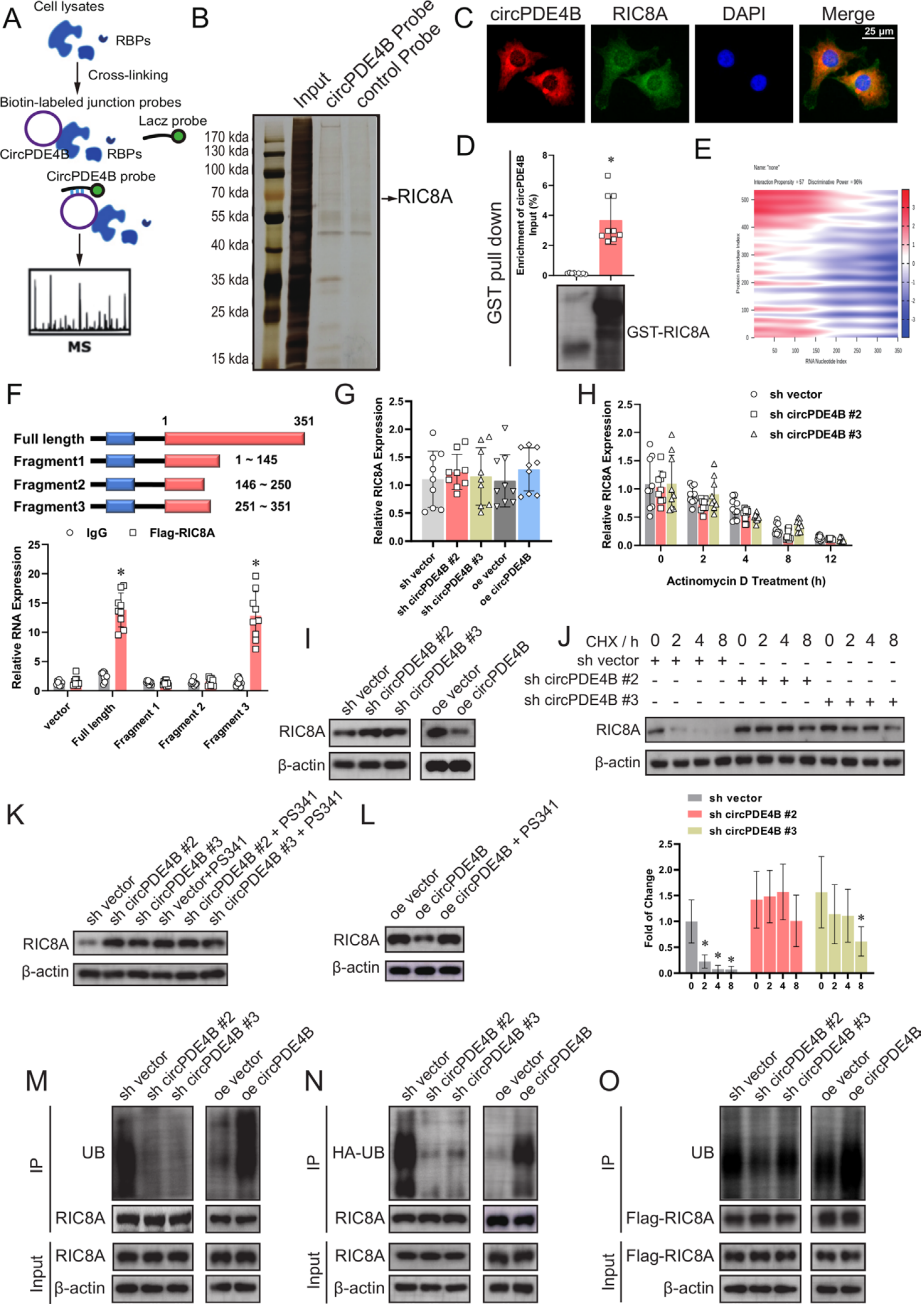
circPDE4B interacts with RIC8A and affects RIC8A ubiquitylation. (A) Schematic of RPD-MS experiments. (B) Silver staining of proteins binding to circPDE4B. (C) circPDE4B and RIC8A interaction in human chondrocytes (HCs) confirmed via an RNA-protein colocalisation assay. Scale bars, 50 µm. (D) RIC8A–circPDE4B interaction detected by GST pull-down assays. GST was used as a pull-down control. (E) Predicted binding sites of circPDE4B and RIC8A (catRAPID graph). (F) Binding sequence of circPDE4B for RIC8A identified by an RIP assay (n=9, 3 donors for three replicates); *p≤0.05. mRNA levels (G), mRNA stability (H) and protein levels (I) of RIC8A after circPDE4B knockdown and overexpression (n=9, 3 donors for three replicates); *p≤0.05. (J) Western blot of RIC8A in HCs treated with the transcription inhibitor CHX (200 µg/mL). (K) Effect of PS341 treatment on RIC8A protein level alteration mediated by circPDE4B knockdown. (L) Effects of PS341 treatment on RIC8A protein expression mediated by circPDE4B overexpression. (M) Immunoprecipitation (IP) analysis of ubiquitinated RIC8A in HCs treated with PS341. The lysates of circPDE4B overexpression or knockdown cells were treated with an anti-RIC8A antibody. (N) HCs were infected with HA-UB lentivirus and then treated with PS341. The lysates of circPDE4B overexpression or knockdown cells were treated with an anti-HA antibody. (O) HCs were infected with Flag–RIC8A lentivirus and then treated with PS341. The lysates of circPDE4B overexpression or knockdown cells were treated with an anti-Flag antibody. CHX, cycloheximide; DAPI, 4',6-Diamidino-2-Phenylindole; GST, glutathione-S-transferase; RBPs, RNA binding proteins; RIC8A, RIC8 guanine-nucleotide exchange factor A; RIP, RNA immunoprecipitation; UB, ubiquitination.

To identify proteins that interact with circPDE4B, we employed RPD-MS (figure 3A and online supplemental figure S5A). A total of 112 proteins interacting with circPDE4B were identified (online supplemental table S2 and figure 3B). We selected five of the highest pep _score proteins and verified their role in the regulation of ECM metabolism in HCs by siRNA knockdown. RT-qPCR results revealed that only RIC8A and ENO1 had an obvious effect on regulating MMP13 and COL2A1 (online supplemental figure S5B). However, the RIP assay indicated that only RIC8A binds to circPDE4B (online supplemental figure S5C). We further confirmed the binding of RIC8A and circPde4b by RIP assay in MCs (figure S5D). RNA-protein colocalisation in HCs also verified the interaction between RIC8A and circPDE4B (figure 3C). RPD assay showed that in vitro linearly transcriptional circPDE4B was able to pull down recombinant RIC8A (figure 3D). We then used the catRAPID tool to predict the interacting regions of circPDE4B and RIC8A (figure 3E and online supplemental figure S5E). To identify the predicted binding sites, we truncated the FL circPDE4B into three segments (S1: 1–145 nt, S2: 146–250 nt, S3: 251–351 nt). In line with the prediction, RIP results indicated only FL and S3 were pulled down by RIC8A (figure 3F). Interestingly, the S3 truncation is reflected as a hairpin region 2 loop in the predicted RNA stem-loop structure (online supplemental figure S5F). Taken together, these results indicate that circPDE4B interacts with RIC8A in HCs.

10.1136/annrheumdis-2021-219969.supp6 Supplementary data

To further investigate the function of RIC8A in the ECM metabolism of HCs, we infected HCs with two RIC8A shRNA adenoviruses (online supplemental figure S6A). CCK-8 assay indicated that RIC8A knockdown increased HCs viability (online supplemental figure S6B). Moreover, RIC8A knockdown cells displayed a significant decrease in the expression of MMP3, MMP13 and ADAMTS4 and increased expression of SOX9, COL2A1 (or COL2 protein) and aggrecan (online supplemental figure S6C–F).

10.1136/annrheumdis-2021-219969.supp7 Supplementary data

We also performed gain-of-function experiments (online supplemental figure S6G). RIC8A overexpression decreased the viability of chondrocytes as revealed by CCK-8 assay (online supplemental figure S6H). Besides, the mRNA and protein expression of MMP3, MMP13 and ADAMTS4 were downregulated, while SOX9, COL2A1 (or COL2 protein) and aggrecan were upregulated in RIC8A-overexpressing HCs (online supplemental figure S6I–L). We further performed western blot and RT-qPCR to assess the influence of mmu_RIC8A on ECM metabolism in MCs. RIC8A also impaired ECM anabolic processes in MCs (online supplemental figure S6M, N). These data collectively support inhibition of cell viability and procatabolic effects of RIC8A in chondrocytes.

### circPDE4B regulates RIC8A function through proteasome-mediated degradation

Our further investigation indicated that circPDE4B regulates RIC8A protein levels, however, not mRNA levels or stability (figure 3G–I). We also blocked RIC8A protein synthesis and observed obvious differences in RIC8A protein half-life between sh-negative control (NC) and sh-circPDE4B HCs (figure 3J), suggesting that circPDE4B decreased RIC8A protein stability. Moreover, in MCs, circPde4b also regulated mmu_RIC8A protein levels (online supplemental figure S6O). To confirm whether circPDE4B affects RIC8A function via changes in post-translational modification, we introduced a proteasome inhibitor named PS341. Accordingly, RIC8A was observed non-changed in both circPDE4B overexpression and knockdown cells after treatment with PS341 (figure 3K, L), indicating that circPDE4B regulates RIC8A through proteasomal activity. Consistently, the polyubiquitination of RIC8A decreased following circPDE4B depletion and increased following circPDE4B overexpression, regardless of endogenous or exogenous RIC8A (figure 3M–O). Cumulatively, these results showed that circPDE4B post-translationally impacts the degradation and turnover of RIC8A mediated by the proteasome.

### circPDE4B facilitates the formation of a ternary complex between RIC8A and midline 1 (MID1) that promotes RIC8A degradation

**Figure 4:**
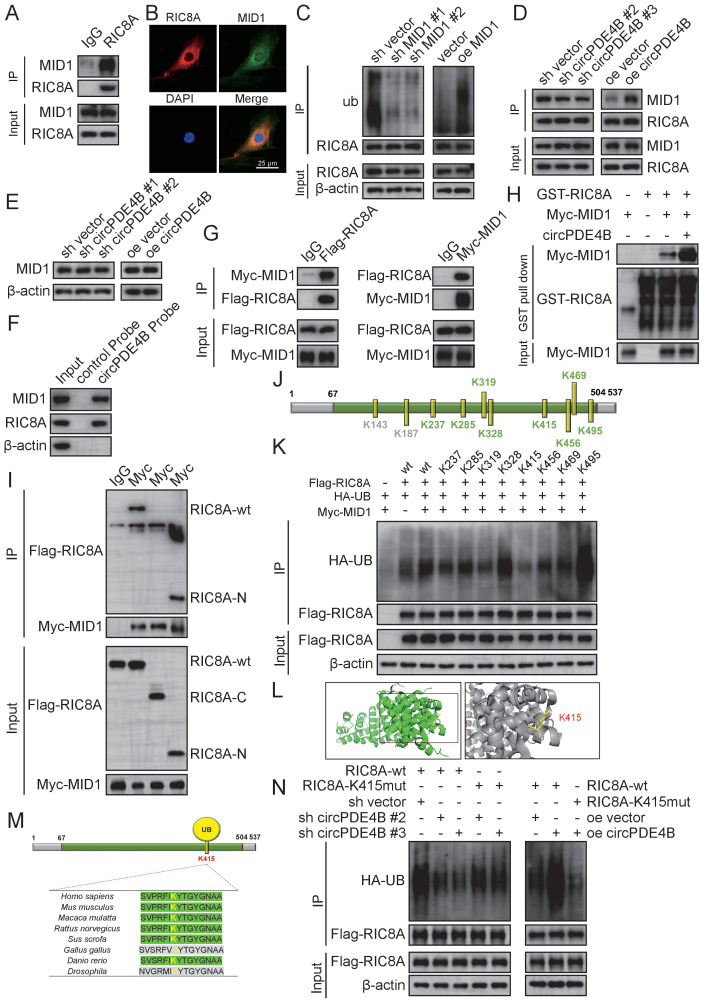
MID1 is an E3 ligase of RIC8A, and K415 is the primary ubiquitylation site of RIC8A. (A) Immunoprecipitation (IP) assay to verify whether E3 ligase MID1 binds to RIC8A. (B) Colocalisation in human chondrocytes (HCs) labelled with anti-RIC8A or anti-MID1 by immunofluorescence. Scale bars, 50 µm. (C) Effect of MID1 overexpression or knockdown on RIC8A ubiquitylation. (D) Effect of circPDE4B overexpression or knockdown on the interaction between RIC8A and MID1. (E) Protein expression of MID1 after circPDE4B knockdown and overexpression. (F) RPD assays using biotin-labelled linear circPDE4B probes in HC lysate before western blotting. (G) HEK-293T cells were infected with Myc–MID1 or Flag–RIC8A before consecutive IP. Rinsing with Flag peptides at first-stage Flag IPs and then eluates were subjected to secondary IP with Myc antibodies or homotypic matching IgG. Western blot was then performed to detect samples. (H) GST–RIC8A and Myc–MID1 overexpressed and purified from cells. RIC8A–MID1 interactions with or without circPDE4B were detected by GST pull-down assays. GST was used as a pull-down control. (I) Myc–MID1 and Flag–RIC8A WT, N-terminal domain and C-terminal domain plasmids were transfected into HEK-293T cells, a co-IP assay was performed and Flag expression was examined by western blotting. (J) HCs were subjected to RIC8A IP and LC–MS/MS analysis of RIC8A ubiquitylation peptide spectra. Ubiquitylated sites were identified by LC–MS analysis. (K) HCs expressing Flag-tagged wild-type or mutant RIC8A KR were first exposed to PS341 and subsequently treated with Flag IP. RIC8A ubiquitylation was analysed via western blot analysis. (L) Crystal structure of RIC8A proteins with K415. (M) Conservation ability of the K415 site of RIC8A. (N) Effect of circPDE4B inhibition and overexpression on the K415R RIC8A ubiquitylation level, as detected by an IP assay. co-IP, co-immunoprecipitation; GST, glutathione-S-transferase; HA-UB, HA-tagged ubiquitination; KR, mutation of lysine (K) to arginine (R); LC, liquid chromatography; MID1, midline 1; MS, mass spectrometry; RIC8A, RIC8 guanine-nucleotide exchange factor A; RPD, RNA pull-down.

We next sought to identify E3 ligases involved in the proteasomal degradation of RIC8A. Interestingly, MS results revealed that circPDE4B also binds two E3 ligases, including RNF2 and MID1. We, therefore, inferred whether circPDE4B could act as a scaffold for RIC8A and E3 ligases complex. However, since RNF2 is localised within the nucleus, we choose MID1 for further investigation. Indeed, MID1 was found to bind RIC8A, as indicated by an immunoprecipitation (IP) assay (figure 4A). Immunofluorescence staining of RIC8A and MID1 also proved their colocalisation in HCs (figure 4B).

Western blot results showed that MID1 decreased RIC8A protein levels (online supplemental figure S7A, B), while IP results indicated that MID1 knockdown effectively impaired the ubiquitylation of RIC8A and MID1 overexpression and increased RIC8A ubiquitylation (figure 4C). Co-immunoprecipitation (Co-IP) assay also revealed that binding of RIC8A and MID1 decreased in circPDE4B knockdown cells compared with control cells, while circPDE4B overexpression had the opposite effect (figure 4D). Moreover, circPDE4B did not affect MID1 levels (figure 4E). Both RPD and sequential IP assays revealed that circPDE4B promotes the binding of RIC8A and MID1 (figure 4F, G). In line with this finding, circPDE4B increased the association between recombinant RIC8A and MID1 proteins in an in vitro binding assay (figure 4H).

10.1136/annrheumdis-2021-219969.supp8 Supplementary data

To further investigate these interactions, we performed domain truncation of RIC8A and MID1 for binding assays. The simple modular architecture research tool (SMART) prediction website indicated that RIC8A contains only a Pfam domain (online supplemental figure S7C). Thus, we divided the protein into two fragments, an N-terminal (1–153 amino acids) and C-terminal (154–537 amino acids) domain. Via co-IP, as expected, MID1 was shown to bind to RIC8A at the N-terminal regulatory domain (figure 4I). In addition, we detected RIC8A functional sites. RIC8A was immunoprecipitated in HCs and subjected to MS analysis, which confirmed ubiquitylation of amino acid residues in RIC8A (figure 4J). Ten ubiquitylation sites were identified in RIC8A, K143 and K187 and were not conserved between humans and mice (figure 4J). We thus mutated conserved RIC8A sites from lysine (K) to arginine (R), to exclude ubiquitylation. IP results indicated that substitution of K415 greatly reduced RIC8A ubiquitylation compared with WT (figure 4K), identifying K415 as the major ubiquitylation site of RIC8A (online supplemental figure S7D). Interestingly, K415 is highly conserved among mammals (figure 4L, M). Further, circPDE4B overexpression or inhibition no longer regulated the ubiquitylation levels of RIC8A following K415 mutation (figure 4N). These results suggest that circPDE4B serves as a scaffold to facilitate the association between RIC8A and MID1.

### circPDE4B and RIC8A regulate the p38 signaling pathway in chondrocytes

**Figure 5:**
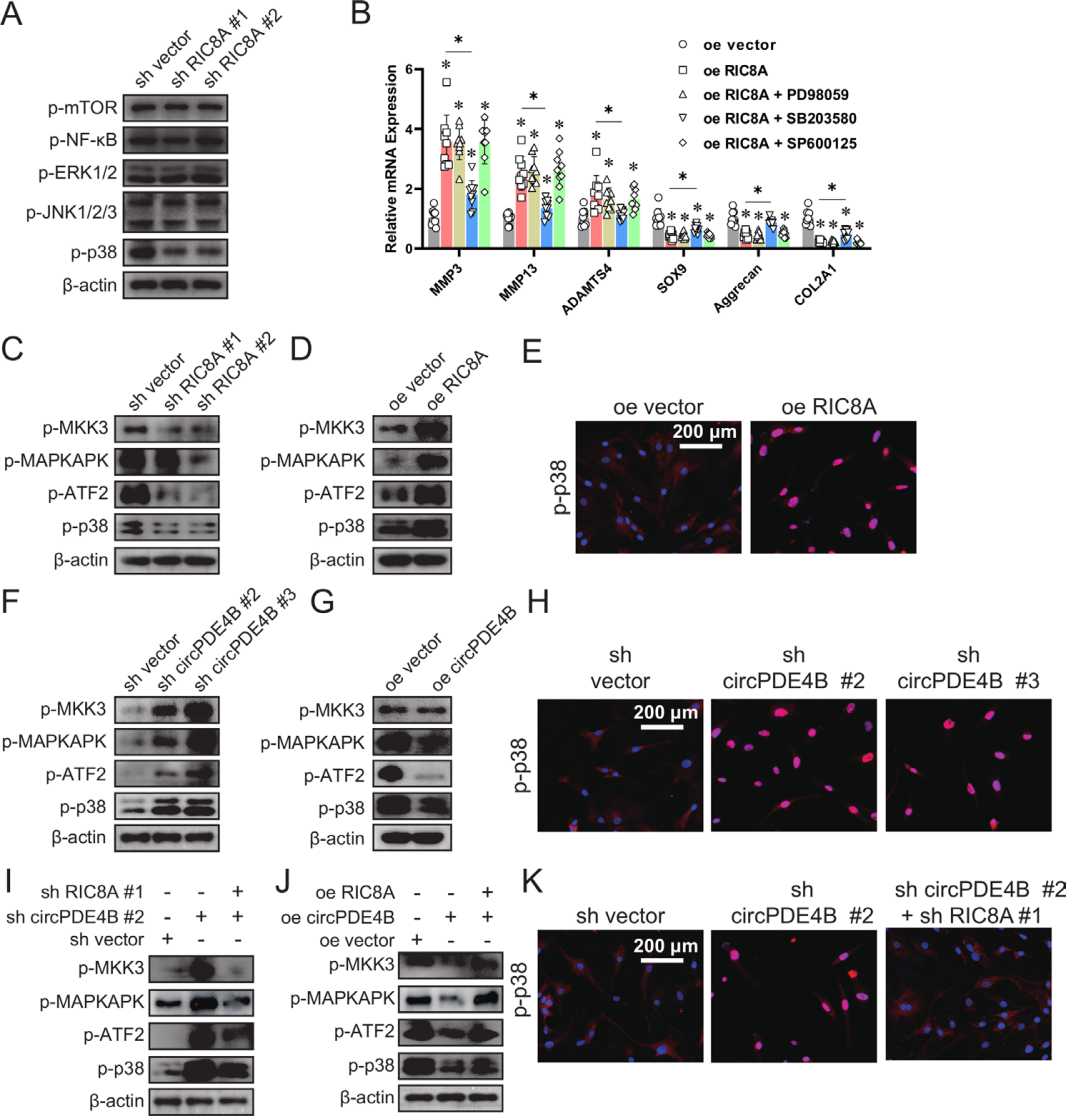
The p38 MAPK pathway is the downstream target of the circPDE4B–RIC8A axis. (A) Phosphorylation of MAPK, NF-κB and mTOR in human chondrocytes (HCs) infected with the vector or RIC8A shRNAs. (B) Relative mRNA expression levels of MMP3/13, ADAMTS4, SOX9, aggrecan and COL2A1 in HCs infected with RIC8A adenovirus and pretreated for 1 hour with PD98059 (ERK inhibitor), SB203580 (p38 MAPK inhibitor) or SP600125 (JNK inhibitor) (n=9, 3 donors for three replicates); *p≤0.05. (C) Phosphorylation levels of p38 MAPK signalling pathway members in HCs infected with RIC8A shRNA or vector adenovirus. Phosphorylation levels of p38 MAPK signal pathway members (D) in HCs with overexpressed RIC8A and (E) associated translocation of p38. (F) Phosphorylation levels of p38 MAPK signalling pathway members in HCs infected with circPDE4B shRNA or vector adenovirus. (G) Phosphorylation levels of p38 MAPK signalling pathway members in HCs infected with circPDE4B adenovirus. (H) Associated translocation of p38 in HCs infected with circPDE4B shRNA or vector adenovirus. (I, J) Phosphorylation levels of p38 MAPK signal pathway members in HCs coinfected with sh circPDE4B and sh RIC8A adenovirus. (I) or circPDE4B and RIC8A overexpression adenovirus. (K) Associated translocation of p38 in HCs coinfected with sh circPDE4B and sh RIC8A adenovirus. ERK, extracellular regulated protein kinases; JNK, c-Jun N-terminal kinase; MAPK, mitogen-activated protein kinase; mTOR, mechanistic target of rapamycin kinase; NF-κB, nuclear factor kappa B; RIC8A, RIC8 guanine-nucleotide exchange factor A.

To elucidate the signalling pathways downstream of RIC8A, we investigated the phosphorylation levels of mitogen-activated protein kinases (MAPKs), NF-κB and mTOR in RIC8A knockdown HCs. The phosphorylation level of p38 was significantly decreased by two RIC8A shRNAs (figure 5A). Next, HCs were pretreated with signalling molecule inhibitors, including PD98059 (extracellular regulated protein kinases 1/2 (ERK1/2) inhibitor), SB203580 (p38 inhibitor) and SP600125 (c-Jun N-terminal kinase (JNK) inhibitor), followed by RIC8A overexpression. The overexpression of RIC8A pretreated with p38 MAPK inhibitors inhibited OA, however, it was not affected by ERK or JNK inhibitors (figure 5B). Moreover, after being infected with RIC8A shRNA or overexpression adenovirus, p38 MAPK phosphorylation and its localisation were dysregulated (figure 5C–E). These results suggest that RIC8A functions through the p38 signalling pathway in chondrocytes.

We next investigated the role of circPDE4B in regulation of the p38 signalling pathway in OA. circPDE4B overexpression decreased while circPDE4B knockdown activated p38 MAPK signalling together with p38 phosphorylation and nuclear translocation (figure 5F–H). We then performed rescue assays. As shown in figure 5I–K, RIC8A overexpression rescued the downregulation of the p38 signalling pathway induced by circPDE4B overexpression, while RIC8A inhibition rescued the activation of p38 signalling pathway induced by circPDE4B knockdown, together with p38 phosphorylation and nuclear translocation. Based on these findings, the circPDE4B–RIC8A axis plays an important role in regulating the downstream p38 MAPK signalling pathway in chondrocytes.

### circPde4b and RIC8A affect OA pathogenesis in mice

**Figure 6:**
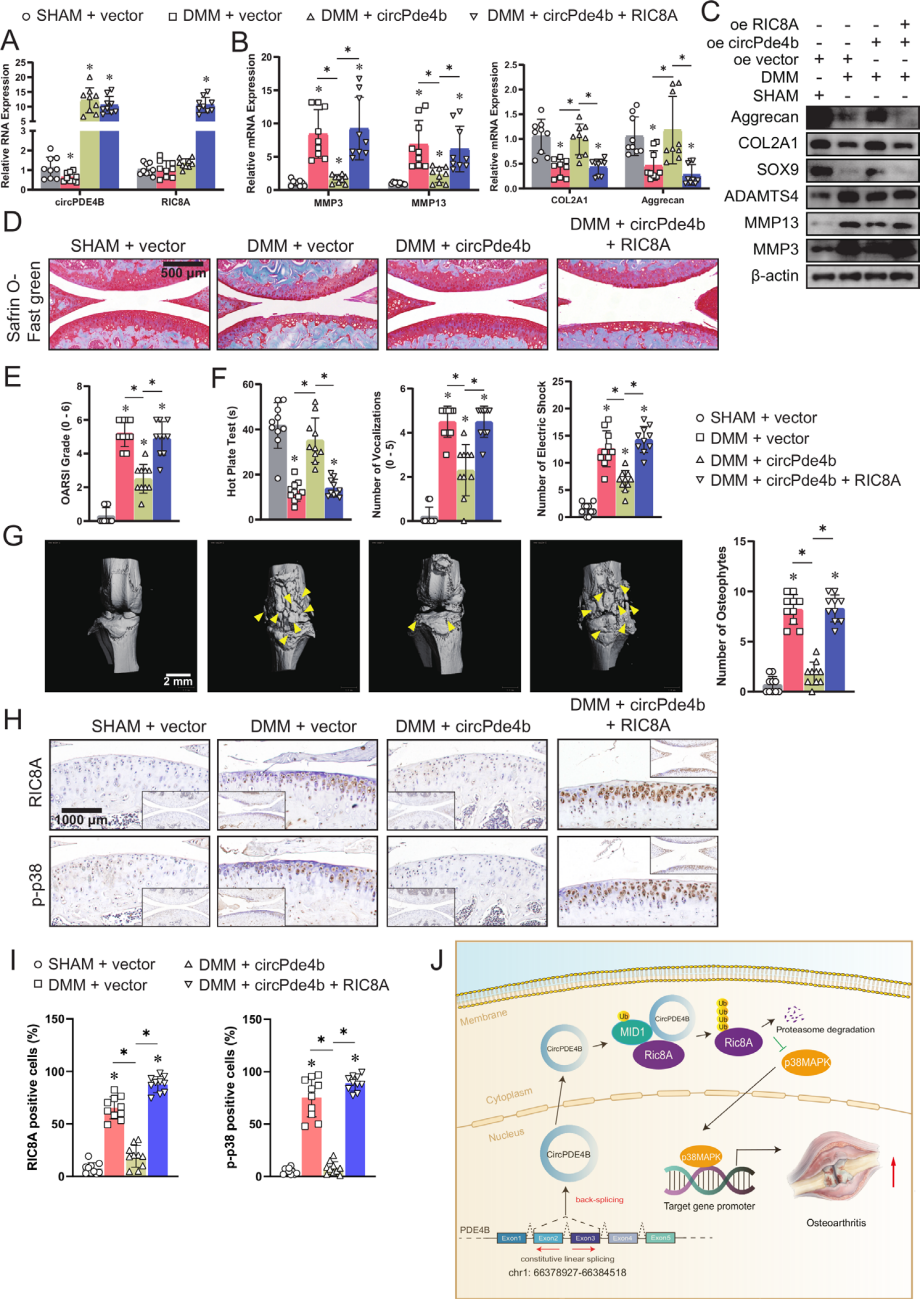
circPDE4B and RIC8A modulates osteoarthritis pathogenesis in a murine model. (A) RT-qPCR quantification of circPDE4B and RIC8A expression in mouse chondrocytes extracted from knee cartilage in the four groups (n=3); *p<0.05. (B) RT-qPCR quantification of MMP3, MMP13, COL2A1 and aggrecan expression in the four groups (n=3); *p<0.05. (C) Western blot analysis of extracellular matrix-associated proteins in the four groups. (D) Representative images of Safranin O fast green staining of cartilage in the four study groups. Scale bars, 500 μm. (E) OARSI grade used for evaluation of the cartilage degradation in the four groups (n=10); *p<0.05. (F) Hot plate test, knee extension test and electric shock-stimulated treadmill test used for the evaluation of knee pain (n=10); *p<0.05. (G) Left, 3D reconstruction images of micro-CT scanning of the knees and osteophytes (yellow arrow). Scale bars, 2 mm. Right, the number of osteophytes (n=10); *p<0.05. (H) Representative images of RIC8A and p-p38-labelled IHC staining. Scale bars, 1000 µm. (I) Quantitative analysis of RIC8A and p-p38 expression in the cartilage with IHC. (n=10); *p<0.05. (J) Graphic abstract of our study. DMM, medialmeniscus destabilisation; IHC, immunohistochemistry; MCs, mouse chondrocytes; OARSI, Osteoarthritis Research Society International; RIC8A, RIC8 guanine-nucleotide exchange factor A. RT-qPCR, quantitative reverse transcription PC.

To corroborate the abovementioned findings, we further assessed the effects of circPde4b on OA in mice (online supplemental figure S8A). The specific adeno-associated virus (AAV) (approximately 1.0×10^10^ vg) efficiently infected the cartilage and synovium in the four groups (online supplemental figure S8B, C), but in vitro study showed that overexpressed circPde4b and RIC8A did not obviously promoted the inflammation of synovium (online supplemental figure S8D, E). Figure 6A shows the RNA expression of circPde4b and RIC8A after infection with the different AAV in the four groups. RT-qPCR and western blot analyses of ECM-associated proteins extracted from cartilage also suggested more severe OA in the medial meniscus destabilisation (DMM)+vector group and DMM+circPde4 b+RIC8A group (figure 6B, C). Using Safranin O fast green staining (figure 6D), marked proteoglycan loss was observed in the DMM+vector group and DMM+circPde4b+RIC8A group compared with the SHAM+vector and DMM+circPDE4B groups, indicating that circPde4b AAV could rescue the OA progression caused by DMM, while RIC8A AAV could reverse this rescue. OARSI grade (figure 6E) further suggested that mice in the SHAM+vector and DMM+circPde4b group displayed less cartilage degradation, whereas those in the DMM+vector and DMM+circPde4b+RIC8A exhibited the opposite. The hot plate test, knee extension test and electric shock stimulated treadmill test demonstrated more discomfort and knee pain in the DMM+vector group and DMM+circPde4b+RIC8A group than in the SHAM+vector and DMM+circPde4b groups (figure 6F). 3D reconstruction of the micro-CT of mouse knees revealed much more osteophytes in the DMM+NC group and DMM+circPde4b+RIC8A group than in the SHAM+vector and DMM+circPde4b groups (figure 6G). MMP3, MMP13, COL2 and aggrecan expression in cartilage from the four groups was consistent with the above staining results (online supplemental figure S9A). Moreover, RIC8A and p-p38 labelled immunohistochemistry (IHC) staining in the four groups showed that overexpressed circPde4b downregulated the RIC8A and p-p38 expression, therefore inhibited the OA progression caused by DMM operation (figure 6H, I). Together, these results indicate that, in mice, circPde4b and RIC8A are involved in OA pathogenesis and their underlying mechanism is presented in figure 6J.

10.1136/annrheumdis-2021-219969.supp9 Supplementary data

10.1136/annrheumdis-2021-219969.supp10 Supplementary data

## Discussion

The OA pathogenesis is primarily underpinned by an imbalance in joint metabolism, for example, when catabolism exceeds anabolism, leading to the degradation of the cartilage matrix.^21^ Emerging evidence has suggested several key catabolic regulators that contribute to cartilage destruction.^22^ However, the mechanism underlying the cessation of matrix anabolism remains largely unknown.

Recent studies have begun to shed light on the various roles of circRNAs including a crucial role in the occurrence, development, diagnosis, prognosis and treatment of diseases.^23^ Specifically, we previously reported that circSERPINE2 could inhibit the occurrence and development of OA by regulating ERG gene as ceRNA.^17^ Zhou *et al* 19 reported a basic role for circRNA33186 in OA development, thus providing a latent drug target for OA therapy. However, when it comes OA, relatively few reports have focused on the importance of circRNAs. Here, we reported that circPDE4B was the most highly expressed among differentially regulated circRNAs obtained through sequencing data. We also observed that circPDE4B is downregulated in chondrocytes treated with IL-1β, as well as the cartilage of OA mice induced by DMM. Further, circPDE4B was inversely related to cartilage degeneration, suggesting that circPDE4B is likely associated with OA development. Further functional experiments revealed that circPDE4B has a key role in OA progression and could represent a therapeutic target.

circRNAs reportedly function through three well-established mechanisms: (1) regulation of parental gene expression and splicing events; (2) complex formation within proteins to perform biological functions and (3) regulating gene expression via miRNA sponging.^24–26^ Herein, we describe the potential mechanism by which circPDE4B can act as a scaffold for RIC8A–MID1 complex, thus promoting RIC8A ubiquitylation. Therefore, we have discovered a distinctive function through which circRNAs can modulate protein stability in OA.

As a guanine nucleotide exchange factor for G-protein alpha subunits, RIC8A was initially identified in *Caenorhabditis elegans*.^27^ RIC8A has been described as an essential protein for G-protein signalling and in centrosome movements during early embryogenesis in *C. elegans*.^28–31^ In mammals, RIC8A disruption in neural progenitors leads to germinal matrix haemorrhage,^32 33^ suggesting that RIC8A activation may represent a key event in human OA pathogenesis. The role of RIC8A in OA, however, remains unclear. Herein, we found that RIC8A plays an important role in OA pathogenesis by regulating p38 MAPK signalling. Previous reports have indicated that the activation of p38, ERK and JNK signalling pathways is strongly correlated with OA cartilage damage.^34–36^ Moreover, MAPKs serve as pivotal signalling molecules that participate in the production of matrix metalloproteinases and regulate viability and differentiation of chondrocytes.^37^ Hence, considering that circPDE4B was found to function through the RIC8A–p38 axis, disruption of this pathway may cause dysregulation of cartilage homeostasis.

Post-translational modifications are associated with disease development and may influence protein function, immunogenicity and subcellular localisation.^38–41^ Ubiquitylation, a major post-translational modification, plays an important role in signal transduction, apoptosis and cell proliferation.^42–44^ Herein, we demonstrated that circPDE4B could disrupt the protein stability of RIC8A and possibly functions by regulating RIC8A post-translational modification. However, such modifications of RIC8A have not been previously reported. Interestingly, we found that circPDE4B also binds to an E3 ligand protein MID1 and RIC8A is ubiquitylated by MID1, thus we infer that circPDE4B promotes RIC8A ubiquitylation by acting as a scaffold to facilitate MID1 binding to RIC8A. Besides, employing acetyl-deficient (K→R) mutants, K415 was identified as a major ubiquitylation site of RIC8A and circPDE4B overexpression or inhibition had no effect on RIC8A K415R ubiquitination.

In summary, our research describes a new circRNA mechanism in OA. We demonstrated that circPDE4B could function as a scaffold for protein degradation and play a crucial role in the progression of OA. circPDE4B was found to regulate ECM metabolism and prevent cartilage matrix construction, validating its latent therapeutic influence on OA development in preclinical animal models. Mechanistically, circPDE4B served as a scaffold to facilitate RIC8A–MID1 binding which decreased RIC8A-dependent activation of p38 signal pathway, thus regulating OA progression. Cumulatively, the results of this study provide prospects for developing novel OA therapies by focusing on reducing the imbalance between matrix synthesis and degradation.

## Data Availability

Data are available in a public, open access repository. All data relevant to the study are included in the article or uploaded as supplementary information.
