# Supplementary material for: circPDE4B prevents articular cartilage degeneration and promotes repair by acting as a scaffold for RIC8A and MID1
Source: Ann Rheum Dis. 2021 May 26;80(9):1209–19. doi: 10.1136/annrheumdis-2021-219969 (PMC8372377; doi:10.1136/annrheumdis-2021-219969)
Supplement: Supplementary data [file annrheumdis-2021-219969supp001.pdf]

SUPPLEMENTARY MATERIALS

CircPDE4B prevents articular cartilage degeneration and promotes repair by acting as a scaffold for RIC8A and MID1

Shuying Shen#, Yute Yang#, Panyang Shen#, Jun Ma, Bin Fang, Qingxin Wang, Kefan Wang, Peihua Shi, Shunwu Fan*&Xiangqian Fang*

SUPPLEMENTARY MATERIALS AND METHODS

Human cartilage

The collection and classification of human cartilage samples was approved by the Ethics Committee of Sir Run Run Shaw Hospital (Zhejiang, China). Informed consent was obtained from all participants. Control cartilage was collected from ten amputees without a history of OA (n = 10), and damaged cartilage was obtained from individuals with a history of OA after total knee replacement surgery (n = 10). The cartilage was divided into three groups: normal medial, OA lateral and OA medial. The tibial plateau cartilage without the protection of a meniscus cover comprising an area of approximately 1 cm (width) × 2 cm (length) along the midline of the medial or lateral tibial plateau was defined as the region of interest (ROI). The Kellgren-Lawrence grade (upon preoperative imaging), Outerbridge grade (by visual observation in isolated specimens), and OARSI grade (after histomorphological staining) was exploited to evaluated specimen OA severity. Among them, Outerbrdige and OARSI grading were used to evaluate the cartilage ROI.

Plasmids

BMJ Publishing Group Limited (BMJ) disclaims all liability and responsibility arising from any reliance placed on this supplemental material which has been supplied by the author(s)

Human/mouse circPDE4B linear sequence (351/350 bp) was synthesized and subcloned into pHBAd-circRNA (HanBio., Shanghai, China). For RNA binding protein immunoprecipitation (RIP) assay, linear circPDE4B truncated mutants and wild type (WT) were cloned into the pcDNA3.1 vector. For RIP and immunoprecipitation (IP) assay, human RIC8A cDNA (1,614 bp) was synthesized and its truncations were subcloned into pcDNA3.1 vector. Mutants of RIC8A were prepared with GeneTailorTM Site-Directed Mutagenesis System (Invitrogen) and primers (table S3). Specially, RIC8A K415R was synthesized and subcloned to pcDNA3.1 vector. For HEK-293T co-IP assay, RIC8A cDNA (1614) /N/C terminal and MID1 cDNA (2,004 bp) were synthesized and then subcloned into pLVX-IRES-puro-Flag or pLVX-IRES-blasticidin-Myc (Addgene). For human chondrocytes (HCs) IP/co-IP assay, Myc-MID1 and Flag-RIC8A lentivirus were then packaged. mmu_RIC8A was also synthesized and then subcloned into pLVX-IRES-puro-Flag, further packaged. CircPDE4B-s and circPDE4B-s-del were also synthesized (circPDE4B-s comprises presumed FUS-binging sites on both flanking introns preserved; circPDE4B-s-del resembles circPDE4B-s, but with FUS sites deleted from the surrounding introns) and subcloned into pLVX-puro vector (Addgene), then packaged lentivirus. RIC8A K415R was also subcloned into pAdEasy-EF1-MCS-CMV-GFP vector and packaged adenovirus (Hanbio Co. Ltd, Shanghai, China). For recombinant adenovirus sh hsa/mmu circPDE4B and hsa/mmu universal shRIC8A construction, oligonucleotides with gene targeting sequences (table S3) were used for the cloning of small hairpin RNA (shRNA)-encoding sequences into pAdEasy-U6-CMV-EGFP vector (Hanbio Co. Ltd, Shanghai, China). For lentivirus shFUS construction, oligonucleotides with gene targeting sequences (table S3) were used for the cloning of small hairpin RNA (shRNA)-encoding sequences into pKLO.1 vector (addgene) and then packaged. HA-ubiquitin (Ubc) lentivirus and control virus were purchased from Hanbio Co. Ltd, Shanghai, China.

Chondrocyte culture

Human chondrocytes (HCs) were extracted from knee cartilage of amputees or subjects after total knee replacement, and mouse chondrocytes (MCs) were obtained from knee cartilage of 3-day-old C57BL/6. Chondrocytes were cultured in Dulbecco’s modified eagle medium (DMEM) supplemented with 10% FBS (Thermo Fisher Scientific, Waltham, MA, USA) within 24 h at 37 °C. Before culture or mRNA isolation, cells were leached using the 0.075 mm filter and rinsed with sterile phosphate-buffered saline (PBS). Primary chondrocytes with 70–80% fusion were used in subsequent experiments. While culturing, cells were cultured in an environment with 5% CO_2 at 37 °C

Animal models

Adult male C57BL/6 mice (n = 40), eight weeks of age, were used for *in vivo* experiments. According to a previous study[1 2], medial meniscus destabilization (DMM) surgery was performed to induce post-traumatic OA to form the positive control. We randomly divided mice into four groups with ten mice per group: SHAM + vector, DMM + vector, DMM + circPde4b, and DMM + circPde4b + RIC8A groups. Briefly, mice (n = 10 per group) were anesthetized, and their medial joint capsules were incised to expose the medial meniscotibial ligament (MMTL). Subsequently, the MMTL was transected with microsurgical scissors to release the ligament linked to the tibial plateau, consequently destabilizing the medial meniscus. The joints were closed after being rinsed with sterile saline. At the same time, the medial knee joint capsule was incised for sham operation. HanBio (Shanghai, China) created and packaged adeno-associated virus (AAV) vectors for circPde4b and RIC8A. One week after operation, a total of 10 μL (approximately 1×10^{12} vg/mL) of vector AAV, circPde4b AAV, or a mixture including 5 μL circPde4b AAV and 5 μL RIC8A AAV was delivered intra-articularly into the knee

joints. Eight weeks later, knee pain was evaluated using a series of assessments[3], including a hot plate test, knee extension test, and electric shock stimulated treadmill test. Briefly, in the 55 °C hot plate test, latency was recorded when the hind paw of the mouse was lifting and/or flicking/licking. In the knee extension test, the number of vocalizations that occurred during five extensions was recorded. For the electric shock stimulated treadmill test, the number of electric shocks stimulating the mouse was recorded within 2 min at a speed of 50 m/min. Following the assessments, mice were sacrificed, and both knee articular cartilages were harvested for histological analysis or gene expression analyses. We conducted all animal experiments following the approval of the Institute of Health Sciences Institutional Animal Care and Use Committee (Zhejiang, China).

Immunofluorescence

Human osteoarthritis chondrocytes were fixed in 4% paraformaldehyde for 30 min, covered with PBS containing 0.25% Triton X-100 for 15 min, and sealed with 5% BSA containing 0.25% Triton X-100 at RT for 30 min. Cells were then incubated with the following primary antibodies: anti-MMP13 (18165-1-AP, proteintech, 1:50), anti-MMP3 (ab52915, abcam, 1:100), anti-COL2 (ab34712, abcam, 1:100), and anti-aggrecan (13880-1-AP, proteintech, 1:50). After overnight incubation at 4 °C, the cells were washed three times with PBS and incubated with goat anti-rabbit IgG (H&L) at room temperature for 1 h, rinsed and the nuclei were restained with DAPI for 5 min. Target proteins were visualized by fluorescence microscopy (Carl Zeiss, USA).

Micro-CT analysis

After fixation in 70% ethanol, all samples were scanned in a 16 mm scanning tube with 10.5 mm3 volume at 55,000 V, 180 mA, with a 115 min acquisition time. To avoid

dehydration, joints were wrapped in napkins dampened with PBS while scanning. The data were analyzed using Skyscan software.

In vitro transcription

Biotin-labeled oligonucleotide probes targeting junction sites of circPDE4B were synthesized (RiboBio, Guangzhou, China). Linear circPDE4B was in vitro transcribed using Biotin RNA Labeling Mix (Roche) and T7 RNA polymerase, circularized using T4 RNA ligase I, treated with RNase R, and purified with RNeasy Mini kit (Qiagen Inc.).

Histological analysis, scoring system and immunohistochemistry

After fixing in 4% paraformaldehyde, paraffin was used to embed cartilage specimens. All samples were cut into 5 μm sections, and each one-tenth was stained with 0.1% Safranin O solution as well as 0.001% Fast Green solution (Sigma-Aldrich, St. Louis, MO, USA). OARSI grade[4 5] was used to grade the severity of cartilage degeneration by two observers blinded to group-identifying information. For human cartilage, the ROI was evaluated using OARSI, while the OA severity of mice cartilage was recorded with the maximal score of observed cartilage.

For immunohistochemistry, the sections were incubated with primary antibodies at 4 °C overnight. Subsequently, the sections were incubated with secondary antibodies for 1 h (Beyotime Institute of Biotechnology, Inc., Jiangsu, China) at 25 °C. All positively stained cells along the joint surface of each sample were counted in the femoral ankle and tibial plateau region, and the proportion of positive cells was evaluated using Image-Pro Plus 6.0 [6].

RNA extraction and RT-qPCR analysis

Total RNA was extracted using RNAEX reagent (Accurate Biotechnology, Hunan) from cultured or isolated chondrocytes. Reverse transcription of mRNAs to cDNA was performed using total RNA, with kits from Accurate Biotechnology (Hunan, China). Specific circRNAs or mRNAs were quantified with SYBR® Green Premix Pro Taq HS qPCR kit (Accurate Biotechnology, Hunan, China). The ABI 7500 Sequencing Detection System (Applied Biosystems, Foster City, CA, USA) was employed for amplification. To obtain circRNA, using 3 U/μg RNase R (Epicenter, San Diego, CA, USA) or not covered total RNA at 37 °C within 20 min. The RNeasy MinElute cleanup kit (Qiagen) was then used to purify RNA. Specific primers were designed to amplify circPDE4B. Agarose gel electrophoresis and sequencing were used to detect amplification products. All reactions were repeated three times. Housekeeping β-actin gene were used for standardization. All primers are listed in table S3.

Co-immunoprecipitation (co-IP)

Co-IP was performed as previously described (Jiao et al, 2018; Li et al, 2018b), with antibodies (1:100 dilution) specific for Myc (Abcam Inc), HA (Cell Signaling Technology), FLAG (Abcam Inc), anti-Ubiquitin antibody (Abcam Inc), RIC8A (Abcam Inc), or MID1 (Abcam Inc). Bead-bound proteins were released and analyzed by western blot.

RNA interference

siRNA-mediated knockdown was used to inhibit circRNA expression. Three different siRNAs (RiboBio, Guangzhou, China) were designed and examined for circPDE4B knockdown efficiency. SiRNA sequences are listed table S3. A specific analog (RiboBio, Guangzhou, China) was used to induce miRNA expression. Plasmid transfection was performed using Lipofectamine 3000 transfection reagent (ThermoFisher). The

Lipofectamine RNAiMAX transfection reagent (ThermoFisher) was applied for siRNA delivery.

RNA binding protein immunoprecipitation

RIP assay was performed using Magna RIPTM RNA Binding Protein Immunoprecipitation kit (Millipore), with antibodies (1:100 dilution) specific for FUS (Abcam Inc), Flag (Abcam Inc) or RIC8A (Abcam Inc). Co-precipitated RNAs were then extracted with TRIzol, and the amount of circPDE4B in the eluate was determined by RT-qPCR (table S3).

RNA pull-down assay and Mass Spectrometry (MS)/ qRT-PCR

The RNA pull-down kit (BersinBio) was used for the RNA pull-down assay. The assay is based on circRNA hybridization with the target-specific biotinylated probe, followed by pull-down, reverse transcription to cDNA for amplification through quantitative real-time PCR (RT-qPCR), and sequencing. After washing with PBS, a total of 10^7 HCs were exposed to ultraviolet irradiation at 254 nm, followed by lysis with 1 mL lysis buffer and homogenized with a 0.4 mm injector. A biotinylated antisense probe (0.2 nmol), which targets the adapter sequence, or Lac Z probes (control probes) were then added to the circPDE4B-RAP mixture. Incubation at 65 °C for 10 mins was carried out, followed by hybridization for 2 h at room temperature. Thereafter, 200 μL of streptavidin-coated magnetic beads was added. Washing followed to remove non-specific binding. Protein digestion yielded peptide fragments, which were then dissolved and vortexed. We used a loading tube to load the supernatants for MS identification. Probes are displayed in table S3.

***In vitro* binding assay**

Shen S, *et al. Ann Rheum Dis* 2021; 80:1209–1219. doi: 10.1136/annrheumdis-2021-219969

For RIC8A GST pull-down assays, GST fusion proteins from *E. coli* BL21 cells were purified overnight at 4 °C and fixed on glutathione Sepharose 4B columns. HEK-293T cells transfected with Flag-MID1 were treated as indicated. Cells were then lysed in the NETN buffer with the protease inhibitor and cultured with Sepharose, and immobilized with indicated RIC8A GST protein and circPDE4B at 4 °C for 8 h. The RIC8A/MID1-circPDE4B complex were pulled down using GST beads (Sigma). Protein was detected by SDS–PAGE and western blot.

Western blot

SDS -PAGE separated total cellular protein, which was then transferred to an Immobilon-P membrane (0.2 μm pore size, Millipore, Billerica, MA, USA). The membranes were blocked and then incubated with primary antibodies (BD Biosciences, San Jose, CA, USA) overnight at 4 °C, and subsequently incubated with secondary antibodies for 1 h. β-actin served as the internal standard and Image J was used to quantify blots intensities. The antibodies used were as follows: anti-MMP13 antibody (1:1000, abcam, ab51072), anti-MMP3 antibody (1:1000, abcam, ab52915), anti-ADAMTS4 antibody (1:1000, abcam, ab185722), anti-COL2 antibody (1:1000, abcam, ab34712), anti-SOX9 antibody (1:1000, abcam, ab185966), anti-aggrecan antibody (1:1000, abcam, ab3778), anti-RIC8A antibody (1:1000, abcam, ab194941), anti-MID1 antibody (1:1000, abcam, ab70770), anti-HA antibody (1:2000, Cell Signaling Technology, 4970s), anti-FLAG antibody (1:1000, abcam, ab205606), anti-Ubiquitin antibody (1:1000, abcam, ab140601), anti-Myc antibody (1:1000, abcam, ab32) and anti-β-actin antibody (1:2000, Cell Signaling Technology, 4970s).

RNA fluorescent *in situ* hybridization (FISH)

The cells were seeded in 12-well plates with sterile glass covers and allowed to culture overnight. Cells were then fixed with PBS containing 37% formaldehyde for 15 min at 25 °C and dehydrated with 70% ethanol at 4 °C for 1 h. Slides were hybridized for 14–16 h at 37 °C. Cy3-labeled circPDE4B probes were constructed by RiboBio (Guangzhou, China). Hybridization buffer for RNA FISH dissolved the probes at a concentration of 20 nM. Following overnight hybridization, the slides were washed with 10% formamide/2× SSC at 37 °C on an oscillator for 30 min, and subsequently washed with PBS and 0.1% (v/v) Tween 20 three times. Cells were then stained with Alexa Fluor 546-conjugated streptavidin for 1 h at the same temperature as for IF. The cover glass was restained with DAPI, fixed with ProLong Gold anti-fading reagents, and observed under a Nikon A1Si Laser Scanning Confocal Microscope (Nikon Instruments Inc., Japan). For FISH of samples from in vivo experiments, ahead of hybridization, we performed the biopsy at 37° C under 0.8% pepsin processing. Slices were then deparaffinized, rehydrated, and permeated for 30 min. The primers and probe sequences are shown in table S3.

Patient and public involvement statement

Patients or the public WERE NOT involved in the design, or conduct, or reporting, or dissemination plans of our research

Statistical analysis

All statistical analyses were performed using SPSS v22.0. Data distribution was assessed using the Shapiro-Wilk test. The Levene test was used to assess the equality of variances. Statistical analysis was performed by unpaired two-tailed Student's t-test (normal distribution and equal variances, using 95% confidence intervals for between-group differences), Welch t-test (unequal variances), or Mann–Whitney U test (non-normal distribution). Multiple group comparisons were performed by one-way analysis of

variance (normal distribution) or Kruskal–Wallis (non-normal distribution) test followed by Bonferroni or Dunn post hoc test, respectively. A P value < 0.05 was considered statistically significant.

REFERENCES

1. Glasson SS, Blanchet TJ, Morris EA. The surgical destabilization of the medial meniscus (DMM) model of osteoarthritis in the 129/SvEv mouse. *Osteoarthritis Cartilage* 2007;15(9):1061-9. doi: 10.1016/j.joca.2007.03.006
2. Chen D, Shen J, Zhao W, et al. Osteoarthritis: toward a comprehensive understanding of pathological mechanism. *Bone Res* 2017;5:16044. doi: 10.1038/boneres.2016.44 [published Online First: 2017/02/06]
3. Piel MJ, Kroin JS, van Wijnen AJ, et al. Pain assessment in animal models of osteoarthritis. *Gene* 2014;537(2):184-8. doi: 10.1016/j.gene.2013.11.091
4. Glasson SS, Chambers MG, Van Den Berg WB, et al. The OARSI histopathology initiative - recommendations for histological assessments of osteoarthritis in the mouse. *Osteoarthritis Cartilage* 2010;18 Suppl 3:S17-23. doi: 10.1016/j.joca.2010.05.025 [published Online First: 2010/10/01]
5. Pritzker KP, Gay S, Jimenez SA, et al. Osteoarthritis cartilage histopathology: grading and staging. *Osteoarthritis Cartilage* 2006;14(1):13-29. doi: 10.1016/j.joca.2005.07.014
6. Chen P, Xia C, Mei S, et al. Intra-articular delivery of sinomenium encapsulated by chitosan microspheres and photo-crosslinked GelMA hydrogel ameliorates osteoarthritis by effectively regulating autophagy. *Biomaterials* 2016;81:1-13. doi: 10.1016/j.biomaterials.2015.12.006 [published Online First: 2015/12/30]

SUPPLEMENTARY TABLES

Table S1. Top 50 differentially expressed circRNAs in control and OA tissues ranked by expression level.

id	log2FC(Con-trol/OA)	pval	regulate	significant	mean_Con	mean_OA
1:65913245|65918835	2.298017962	0.013420134	up	yes	71.57389833	14.47433303

2:223998115|224001922	2.017322781	0.037510887	up	yes	57.89743315	14.2263021
6:159682474|159688242	3.277353459	0.000604761	up	yes	38.51538065	3.882709433
7:84129123|84194668	4.416423377	0.000404841	up	yes	23.89613671	1.023738907
4:102304317|102315830	3.801482647	0.003360458	up	yes	21.73943548	1.4663222
2:223991803|224001922	2.334035326	0.013161838	up	yes	18.03811377	3.497306791
13:42953948|42970670	2.907556748	0.009637056	up	yes	16.17980657	2.069638833
5:65170617|65197151	4.154129743	0.015296915	up	yes	15.86773823	0.796862253
17:27765535|27766588	7.209593338	2.94E-10	up	yes	14.70143599	0
1:102979382|102989571	7.01468859	9.33E-11	up	yes	12.83098702	0
1:32948222|32949774	1.899306098	0.048974712	up	yes	7.811225125	2.020780085
8:17558295|17562110	5.830035008	7.60E-07	up	yes	7.605289257	0.035448292
19:45025329|45025737	2.667894244	0.014344495	up	yes	6.992268417	1.016012274
7:80761616|80789528	2.166891001	0.025288244	up	yes	6.740826549	1.423384001
19:6702127|6702579	5.725610351	3.17E-08	up	yes	5.191520209	0
8:17543318|17543715	4.576294017	1.42E-05	up	yes	4.946360357	0.111532203
5:59180595|59215968	5.27607838	1.18E-07	up	yes	3.774876381	0
10:7243558|7285954	1.954291245	0.049795018	up	yes	3.444247889	0.814584431
17:27768977|27769584	5.136722549	0.000186202	up	yes	3.418095033	0
20:54157169|54171670	4.243602481	8.27E-05	up	yes	3.089979272	0.068397727
13:42917541|	3.094148388	0.011943706	up	yes	2.836359073	0.243856855

42970670						
7:84110470|84194668	4.361466685	0.000348323	up	yes	2.810081997	0.041570553
6:107503657|107533610	2.581974853	0.045526372	up	yes	2.492951616	0.333054478
20:18297985|18306393	4.687371106	0.002144755	up	yes	2.476554324	0
4:88475841|88479507	4.543128739	4.12E-06	up	yes	2.231406616	0
7:84046324|84060558	4.025668711	0.012672298	up	yes	2.160629655	0.038797737
7:90747681|90790652	4.475075842	3.16E-06	up	yes	2.123986063	0
1:27668226|27669346	3.850469968	0.001064668	up	yes	2.109467326	0.053172438
17:66513336|66514466	4.390178942	0.001302459	up	yes	1.996889514	0
18:6301903|6312056	2.876917257	0.039095578	up	yes	1.93391171	0.176881352
1:186893011|186911389	3.106673582	0.00897813	up	yes	1.899981712	0.132179616
18:6263947|6312056	3.497797506	0.00378226	up	yes	1.830524648	0.070896584
X:148661908|148662768	2.136810258	0.026904799	up	yes	1.829022055	0.338624828
3:112109543|112113145	3.347239351	0.003786519	up	yes	1.827445988	0.089392497
4:150467673|150491035	4.255549433	1.27E-05	up	yes	1.810064475	0
17:27778690|27779056	4.225978288	1.16E-05	up	yes	1.771312099	0
22:33761371|33765953	4.194653636	0.001595289	up	yes	1.731118987	0
17:80289659|80290728	2.289891436	0.029722666	up	yes	1.684744047	0.26496392
8:105419144|105561481	2.399627165	0.025199407	up	yes	1.584695203	0.219272552
19:45022053|45028992	3.28409336	0.006756638	up	yes	1.569980322	0.071435494

2:223912253|223918010	4.053146681	0.000134317	up	yes	1.560040669	0
13:42926336|42970670	2.181505031	0.034446179	up	yes	1.511971175	0.255352047
15:45476079|45476552	3.959830453	0.001227535	up	yes	1.456065035	0
1:46077515|46080750	3.950560938	8.63E-05	up	yes	1.446099152	0
5:65170617|65215487	3.94001855	6.71E-05	up	yes	1.434842326	0
5:65242104|65273447	2.83649554	0.031810662	up	yes	1.431521832	0.114413908
14:100353687|100361921	2.884414792	0.048709863	up	yes	1.424099749	0.106403957
17:80325030|80328477	3.013454971	0.006302126	up	yes	1.419825328	0.088214614
7:84110470|84134951	3.482653818	0.002962863	up	yes	1.414107802	0.035448292
1:102939035|102946956	2.892345557	0.03182733	up	yes	1.300464786	0.088620731

Table S2. Top 50 circPDE4B-binding proteins identified by mass spectrometry (ranked by pep_score).

num	prot_acc	prot_desc	pep_score
1	P07237	Protein disulfide-isomerase OS=Homo sapiens OX=9606 GN=P4HB PE=1 SV=3	118.07
2	Q9NPQ8	Synembryn-A OS=Homo sapiens OX=9606 GN=RIC8A PE=1 SV=3	96.92
3	P06733	Alpha-enolase OS=Homo sapiens OX=9606 GN=ENO1 PE=1 SV=2	95.8
4	Q16891	MICOS complex subunit MIC60 OS=Homo sapiens OX=9606 GN=IMMT PE=1 SV=1	95.59
5	P04259	Keratin, type II cytoskeletal 6B OS=Homo sapiens OX=9606 GN=KRT6B PE=1 SV=5	92.43

6	P67936	Tropomyosin alpha-4 chain OS=Homo sapiens OX=9606 GN=TPM4 PE=1 SV=3	90.97
7	P08133	Annexin A6 OS=Homo sapiens OX=9606 GN=ANXA6 PE=1 SV=3	87.9
8	Q6WCQ1	Myosin phosphatase Rho-interacting protein OS=Homo sapiens OX=9606 GN=MPRIP PE=1 SV=3	84.9
9	P27348	14-3-3 protein theta OS=Homo sapiens OX=9606 GN=YWHAQ PE=1 SV=1	83.69
10	O00458	Interferon-related developmental regulator 1 OS=Homo sapiens OX=9606 GN=IFRD1 PE=1 SV=4	82.38
11	P04181	Ornithine aminotransferase, mitochondrial OS=Homo sapiens OX=9606 GN=OAT PE=1 SV=1	81.54
12	Q9UNL2	Translocon-associated protein subunit gamma OS=Homo sapiens OX=9606 GN=SSR3 PE=1 SV=1	80.32
13	P10809	60 kDa heat shock protein, mitochondrial OS=Homo sapiens OX=9606 GN=HSPD1 PE=1 SV=2	77.24
14	Q15084	Protein disulfide-isomerase A6 OS=Homo sapiens OX=9606 GN=PDIA6 PE=1 SV=1	72.89
15	Q7Z406	Myosin-14 OS=Homo sapiens OX=9606 GN=MYH14 PE=1 SV=2	71.8
16	E9PRG8	Uncharacterized protein C11orf98 OS=Homo sapiens OX=9606 GN=C11orf98 PE=4 SV=1	71.22
17	P68871	Hemoglobin subunit beta OS=Homo sapiens OX=9606 GN=HBB PE=1 SV=2	67.41
18	P83731	60S ribosomal protein L24 OS=Homo sapiens OX=9606 GN=RPL24 PE=1 SV=1	66.31
19	P21333	Filamin-A OS=Homo sapiens OX=9606 GN=FLNA PE=1 SV=4	66.06
20	P21127	Cyclin-dependent kinase 11B OS=Homo sapiens OX=9606	65.88

		GN=CDK11B PE=1 SV=4	
21	P48444	Coatomer subunit delta OS=Homo sapiens OX=9606 GN=ARCN1 PE=1 SV=1	64.61
22	Q12965	Unconventional myosin-Ie OS=Homo sapiens OX=9606 GN=MYO1E PE=1 SV=2	62.35
23	P42766	60S ribosomal protein L35 OS=Homo sapiens OX=9606 GN=RPL35 PE=1 SV=2	61.42
24	P05783	Keratin, type I cytoskeletal 18 OS=Homo sapiens OX=9606 GN=KRT18 PE=1 SV=2	60.52
25	P05783	Keratin, type I cytoskeletal 18 OS=Homo sapiens OX=9606 GN=KRT18 PE=1 SV=2	60.52
26	P10412	Histone H1.4 OS=Homo sapiens OX=9606 GN=HIST1H1E PE=1 SV=2	58.75
27	P43246	DNA mismatch repair protein Msh2 OS=Homo sapiens OX=9606 GN=MSH2 PE=1 SV=1	57.6
28	O43707	Alpha-actinin-4 OS=Homo sapiens OX=9606 GN=ACTN4 PE=1 SV=2	57.2
29	P08865	40S ribosomal protein SA OS=Homo sapiens OX=9606 GN=RPSA PE=1 SV=4	56.12
30	Q9NX58	Cell growth-regulating nucleolar protein OS=Homo sapiens OX=9606 GN=LYAR PE=1 SV=2	55.3
31	P06396	Gelsolin OS=Homo sapiens OX=9606 GN=GSN PE=1 SV=1	55.09
32	Q9Y4I1	Unconventional myosin-Va OS=Homo sapiens OX=9606 GN=MYO5A PE=1 SV=2	54.65
33	P62937	Peptidyl-prolyl cis-trans isomerase A OS=Homo sapiens OX=9606 GN=PPIA PE=1 SV=2	54
34	Q15233	Non-POU domain-containing octamer-binding protein OS=Homo sapiens OX=9606 GN=NONO PE=1 SV=4	51.87

35	Q7KZF4	Staphylococcal nuclease domain-containing protein 1 OS=Homo sapiens OX=9606 GN=SND1 PE=1 SV=1	51.86
36	Q5BJD5	Transmembrane protein 41B OS=Homo sapiens OX=9606 GN=TMEM41B PE=1 SV=1	50.71
37	P13796	Plastin-2 OS=Homo sapiens OX=9606 GN=LCP1 PE=1 SV=6	48.92
38	Q02809	Procollagen-lysine,2-oxoglutarate 5-dioxygenase 1 OS=Homo sapiens OX=9606 GN=PLOD1 PE=1 SV=2	48.83
39	P47755	F-actin-capping protein subunit alpha-2 OS=Homo sapiens OX=9606 GN=CAPZA2 PE=1 SV=3	47.71
40	P62701	40S ribosomal protein S4, X isoform OS=Homo sapiens OX=9606 GN=RPS4X PE=1 SV=2	46.2
41	Q96D15	Reticulocalbin-3 OS=Homo sapiens OX=9606 GN=RCN3 PE=1 SV=1	46.01
42	P62241	40S ribosomal protein S8 OS=Homo sapiens OX=9606 GN=RPS8 PE=1 SV=2	45.16
43	P27797	Calreticulin OS=Homo sapiens OX=9606 GN=CALR PE=1 SV=1	44.29
44	P31946	14-3-3 protein beta/alpha OS=Homo sapiens OX=9606 GN=YWHAB PE=1 SV=3	42.45
45	P04844	Dolichyl-diphosphooligosaccharide--protein glycosyltransferase subunit 2 OS=Homo sapiens OX=9606 GN=RPN2 PE=1 SV=3	42.04
46	P46777	60S ribosomal protein L5 OS=Homo sapiens OX=9606 GN=RPL5 PE=1 SV=3	41.71
47	P14618	Pyruvate kinase PKM OS=Homo sapiens OX=9606 GN=PKM PE=1 SV=4	41.29
48	P60866	40S ribosomal protein S20 OS=Homo sapiens OX=9606 GN=RPS20 PE=1 SV=1	40.91
49	Q9Y281	Cofilin-2 OS=Homo sapiens OX=9606 GN=CFL2 PE=1 SV=1	40.89

Shen S, *et al. Ann Rheum Dis* 2021; 80:1209–1219. doi: 10.1136/annrheumdis-2021-219969

50	Q9UN86	Ras GTPase-activating protein-binding protein 2 OS=Homo sapiens OX=9606 GN=G3BP2 PE=1 SV=2	40.52

Table S3. Primers and sequences used in this study.

Primers for qPCR		
PDE4B mRNA (hsa)	F	CACACTTGGGATCGACCTCT
	R	GGAAGCGTTGTCAAAGGCAG
circPDE4B (hsa)	F	CAGAGTGAAAGGGCAAGGACT
	R	CCGTCATCACACTCCTGCTTT
Pde4b mRNA (mmu)	F	TGCGCTTGGAACTTGAGCTT
	R	GCCGCTGTCGGATGCTTTTA
circPde4b (mmu)	F	CTGTGAGCCAGGAGTGGTCTA
	R	GGTGTAACTGGAAGAGCTGTAGGAT
Human β-actin	F	AGAGCTACGAGCTGCCTGAC
	R	AGCACTGTGTTGGCGTACAG
Mouse β-actin	F	AGCCATGTACGTAGCCATCC
	R	CTCTCAGCTGTGGTGGTGAA
Human MMP3	F	CCTACAAGGAGGCAGGCAAG
	R	CCCGTCACCTCCAATCCAAG
Human MMP13	F	TCGGCCACTCCTTAGGTCTT
	R	AAGTGGCTTTTGCCGGTGTA
Human COL2A1	F	TCCTGCCGTTTCGCTG
	R	CATTATACCTCTGCCCATCCTG
Human ADAMTS4	F	GTCCCATGTGCAACGTCAAG
	R	ATGCGGCCATCTTGTCATCT
Human ADAMTS5	F	GGGCACTGGCTACTATGTGG
	R	CGTCACAGCCAGTTCTCACA
Human Aggrecan	F	GGGACCTGCAAGGAGACAGAG
	R	TCAATCTCACACAGGTCCCCTTC
Human SOX9	F	GCTCTGGAGACTTCTGAACGA
	R	CCGTTCTTCACCGACTTCCT
Human RIC8A	F	GCTCTGCGGTCATACAACCAG
	R	GTCTCTTCCGGTCCTCCTGTT
Mouse RIC8A	F	AGATGCGGTGACAGAAGCTC
	R	CACGGTGCTTTGGTGACAAG
Mouse MMP13	F	CAAGCAGTTCCAAAGGCTACA

	R	TAGGGCTGGGTCACACTTCT
Mouse MMP3	F	ACTGTGTCCCAAGGAGAGGAG
	R	AAACCATCTACACAGTTCAGACAC
Mouse COL2A1	F	CACGCATGAGCCGAAGCTA
	R	GGGTTTCCACGTCTCACCA
Mouse ADAMTS4	F	TTGTTCTCCCAGTCACCCTCC
	R	AGCCTGGGACTAAAGATAGGCA
Mouse ADAMTS5	F	ATGCAGCCATCCTGTTCACC
	R	AAGGCCAAGTAGATGCCCAATTT
Mouse Aggrecan	F	CACTGTCAAAGCACCATGCC
	R	TAGGCTGGCTCCCATTCAGT
Mouse SOX9	F	TAATTCCCCAGGCTCTTGGAT
	R	GCAGCCGGGATTTAAGGCTC
Human MID1	F	CTGATCCGAGGCAAAGAGCC
	R	CAGGCACTCGTCACAGTAGG
Human FUS	F	TTATGGCCAGAGCCAGAACAC
	R	GCTACCGTAACTTCCCGAGG
Human DHX9	F	GTACGGCCTGGATTCTGCTT
	R	ATCACAGCATCCAAAGGGGG
Primers for Site-Directed Mutagenesis		
RIC8A K143R	F	ctctgtgagcctcaccactaggcgggc
	R	gcccgcctagtggtgaggctcacagag
RIC8A K187R	F	gcgcactcctctcagctcctgaaacagctg
	R	cagctgtttcaggagctgagaggagtgcgc
RIC8A K237R	F	acctcccccctgatggagtccagggtg
	R	caccctggactccatcaggggggaggt
RIC8A K285R	F	gagaacatccagacacctgaggggcaagttccc
	R	gggaacttgcccctcaggtgtctggatgttctc
RIC8A K319R	F	tgtgcaaacgcctctctaggaagatgaggaggg
	R	ccctcctcatcttcctagagaggcgtttgcaca
RIC8A K328R	F	ctacactctccctcagcctgtgtgtcttgtgca
	R	tgcacaagacacacaggctgagggagagtgtag
RIC8A K415R	F	cccatagcctgtgtacctgatgaatcggggcac
	R	gtgccccgattcatcaggtacacaggctatggg
RIC8A K456R	F	tttatgctggctctggcttccttgtactcatctgtg
	R	cacagatgagtacaaggaagccagagccagcataaa
RIC8A K469R	F	ttaggcggcctctcctccaccctcccg
	R	cgggagggtggaggagaggccgcctaa

Primers for PCR		
Divergent- β-actin	F	CAGGGCTTACCTGTACACTGA
	R	GCGCGGCGATATCATCATCC
β-actin	F	AGAGCTACGAGCTGCCTGAC
	R	AGCACTGTGTTGGCGTACAG
CircPDE4B	F	CAGAGTGAAAGGGCAAGGACT
	R	CCGTCATCACACTCCTGCTTT
mPDE4B	F	CACACTTGGGATCGACCTCT
	R	GGAAGCGTTGTCAAAGGCAG
Divergent- β-actin (Mouse)	F	GGCCTGTACACTGACTTGAGA
	R	AAGGAGCTGCAAAGAAGCTGT
β-actin (Mouse)	F	AGCCATGTACGTAGCCATCC
	R	CTCTCAGCTGTGGTGGTGAA
CircPDE4B (Mouse)	F	CTGTGAGCCAGGAGTGGTCTA
	R	GGTGTAACTGGAAGAGCTGTAGGAT
PDE4B (Mouse)	F	TGCGCTTGGAACTTGAGCTT
	R	GCCGCTGTCGGATGCTTTTA
ShRNA		
CircPDE4B shRNA-1	F	CCGGCCAGGAGTGGTATTAAAAACTCGAGTTTTTAATACCACTCCTGGTTTTTC
	R	AATTGAAAAACCAGGAGTGGTATTAAAAACTCGAGTTTTTAATACCACTCCTGG
CircPDE4B shRNA-2	F	CCGGGGAGTGGTATTAAAAAGTGCTCGAGCACTTTTTAATACCACTCCTTTTTC
	R	AATTGAAAAAGGAGTGGTATTAAAAAGTGCTCGAGCACTTTTTAATACCACTCC
RIC8A shRNA-1	F	CCGGACCGCACAGAGGAGTTCCACTCGAGTGGAACTCCTCTGTGCGGTTTTTC
	R	AATTGAAAAAGGAGTGGTATTAAAAAGTGCTCGAGCACTTTTTAATACCACTCC
RIC8A shRNA-2	F	CCGGCATGTTTGACAAGCTCTCCCTCGAGGGAGAGCTTGTCAAACATGTTTTTC
	R	AATTGAAAAACATGTTTGACAAGCTCTCCCTCGAGGGAGAGCTTGTCAAACATG
MID1 shRNA-1	F	CCGGACCGCATCCTAGTATCACACTCGAGTGTGATACTAGGATGCGGTTTTTC
	R	AATTGAAAAAACCGCATCCTAGTATCACACTCGAGTGTGATACTAGGATGCGGT

MID1 shRNA-2	F	CCGGGCAACGTCACCCTACAGAACTCGAGTTCTGTAGGGTGACGTTGCTTTTTC
	R	AATTGAAAAAGCAACGTCACCCTACAGAACTCGAGTTCTGTAGGGTGACGTTGC
FUS shRNA-1	F	CCGGCAAGCAGATTGGTATTATTCTCGAGAATAATACCAATCTGCTTGTTTTTC
	R	AATTGAAAAACAAGCAGATTGGTATTATTCTCGAGAATAATACCAATCTGCTTG
FUS shRNA-2	F	CCGGCAGAGTTACAGTGGTTATACTCGAGTATAACCACTGTAACTCTG TTTTTC
	R	AATTGAAAAACAGAGTTACAGTGGTTATACTCGAGTATAACCACTGTAACTCTG
Mmu CircPDE4B shRNA-1	F	CCGGGGAGTGGTCTAATCTGCCACTCGAGTGGCAGATTAGACCACTCCTTTTTC
	R	AATTGAAAAAGGAGTGGTCTAATCTGCCACTCGAGTGGCAGATTAGACCACTCC
Mmu CircPDE4B shRNA-2	F	CCGGGCCAGGAGTGGTCTAATCTCTCGAGAGATTAGACCACTCCTGGCTTTTTC
	R	AATTGAAAAAGCCAGGAGTGGTCTAATCTCTCGAGAGATTAGACCACTCCTGGC
SiRNAs		
Scramble si		UUCUCCGAACGUGUCACGUTT
Human CircPDE4B si-1		AGCCAGGAGTGGTATTAAA
Human CircPDE4B si-2		CCAGGAGTGGTATTAAAAA
Human CircPDE4B si-3		GGAGTGGTATTAAAAAGTG
Mouse CircPDE4B si-1		GGAGTGGTCTAATCTGCCA
Mouse CircPDE4B si-2		GCCAGGAGTGGTCTAATCT
Mouse CircPDE4B si-3		TGAGCCAGGAGTGGTCTAA
FUS si-1		CAAGCAGATTGGTATTATT
FUS si-2		CAGAGTTACAGTGGTTATA
DHX9 si-1		CGAACACCATTGCATGAAA
DHX9 si-2		GGACTAGTAGCAACATTGA
Human/Mouse RIC8A si-1		ACCGCACAGAGGAGTTCCA
Human/Mouse RIC8A si-2		CATGTTTGACAAGCTCTCC
Human KRT6B si		TACCATCAAGTCAACAGTTATCA
Human ENO1 si		GCATTGGAGCAGAGGTTTA

Shen S, et al. Ann Rheum Dis 2021; 80:1209–1219. doi: 10.1136/annrheumdis-2021-219969

Human IMMT si		GGGTTGACTACTGGCAAAATTGC
Human P4HB si		GTCCTCTTTAAGAAGTTTGATGA
Probes for FISH		
Cy3-Hsa circPDE4B		5‘-Cy3-ACACTTTTTAATACCACTCCTGGCTTACAG-3’
Cy3- Mmu circPDE4B		5‘-CY3-GGCAGATTAGACCACTCCTGGCTCAC-3’
RAP and PCR sequences		
CircPDE4B pull-down probe	CATTGCTATTACAACTGTAAGCCAGGAGTGGTATTAAAAAGTGTCAGCAAACTGCATTGAATAACAGACATCCTAAGAGGGGATATTTTCCACCTCTATAATGAAGAAAAGCAGGAGTGTGATGACGGTGATGGCTGATGATAATGTTAAAGATTATTTTGAATGTAGCTTGAGTAAATCCTACAGTTCTTCCAGTAACACACTTGGGATCGACCTCTGGAGAGGGAGAAGGTGTTGCTCAGGAAACTTACAGTTACCACCACTGTCTCAAAGACAGAGTGAAAGGGCAAGGACTCCTGAGGGAGATGGTATTTCCAGGCCGACCACACTGCCTTTGACAACGCTTCCAAGCATTGCTATTACAACTGTAAGCCAGGAGTG	
Lac Z pull-down probes	TGGCCGTCGTTTTACAACGTCGTGACTGGGAAAACCCTGGCGTTACCCAACTTAATCGCCTTGCAGCACATCCCCCTTTCGCCAGCTGGCGTAATAGCGAAGAGGCCCGCACCGATCGCCCTTCCCAACAGTTGCGCAGCCTGAATGGCGAATGGCGCCTGATGCGGTATTTTCTCCTTACGCATCTGTGCGGTATTTCACACCGCATATGGTGCACTCTCAGTACAATCTGCTCTGATGCCGCATAG	
