# Supplementary figures and images for: circPDE4B prevents articular cartilage degeneration and promotes repair by acting as a scaffold for RIC8A and MID1
Source: Ann Rheum Dis. 2021 May 26;80(9):1209–19. doi: 10.1136/annrheumdis-2021-219969 (PMC8372377; doi:10.1136/annrheumdis-2021-219969)

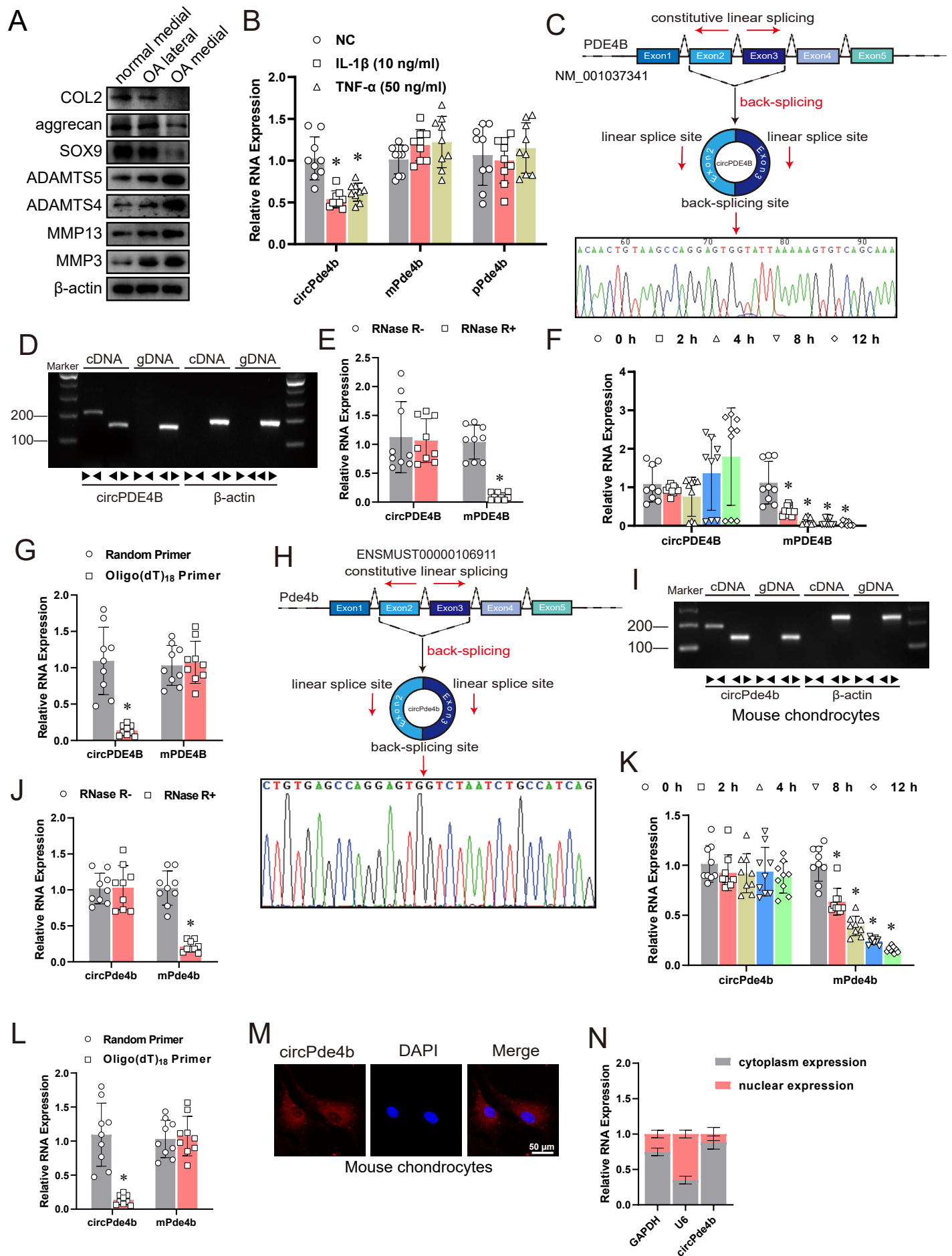

Supplement: Supplementary data [file annrheumdis-2021-219969supp002.pdf]

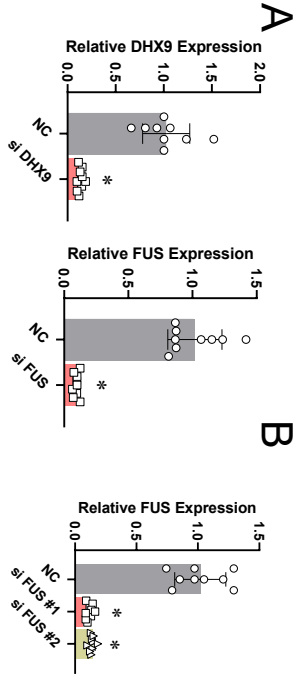

Supplement: Supplementary data [file annrheumdis-2021-219969supp003.pdf]

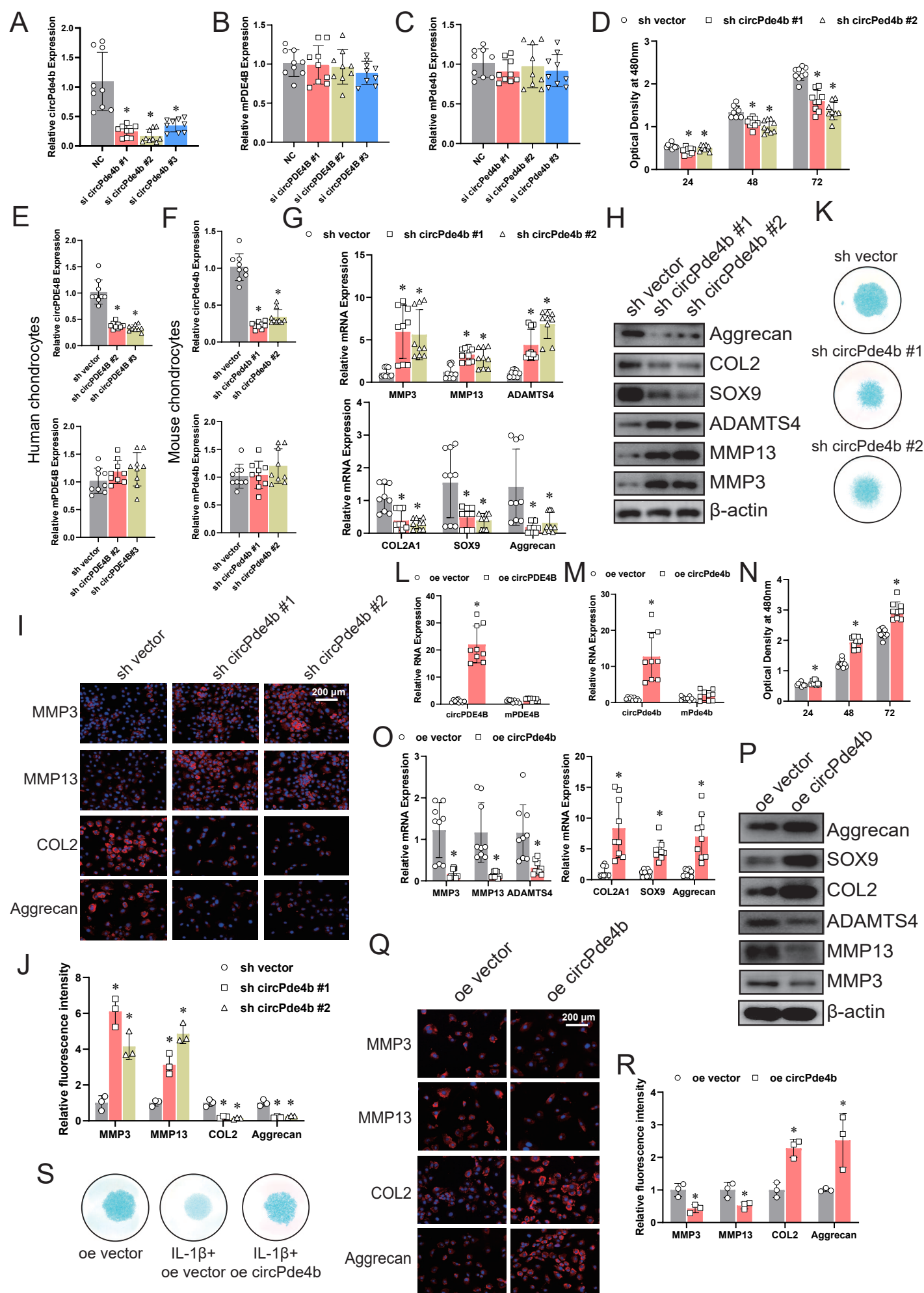

Supplement: Supplementary data [file annrheumdis-2021-219969supp004.pdf]

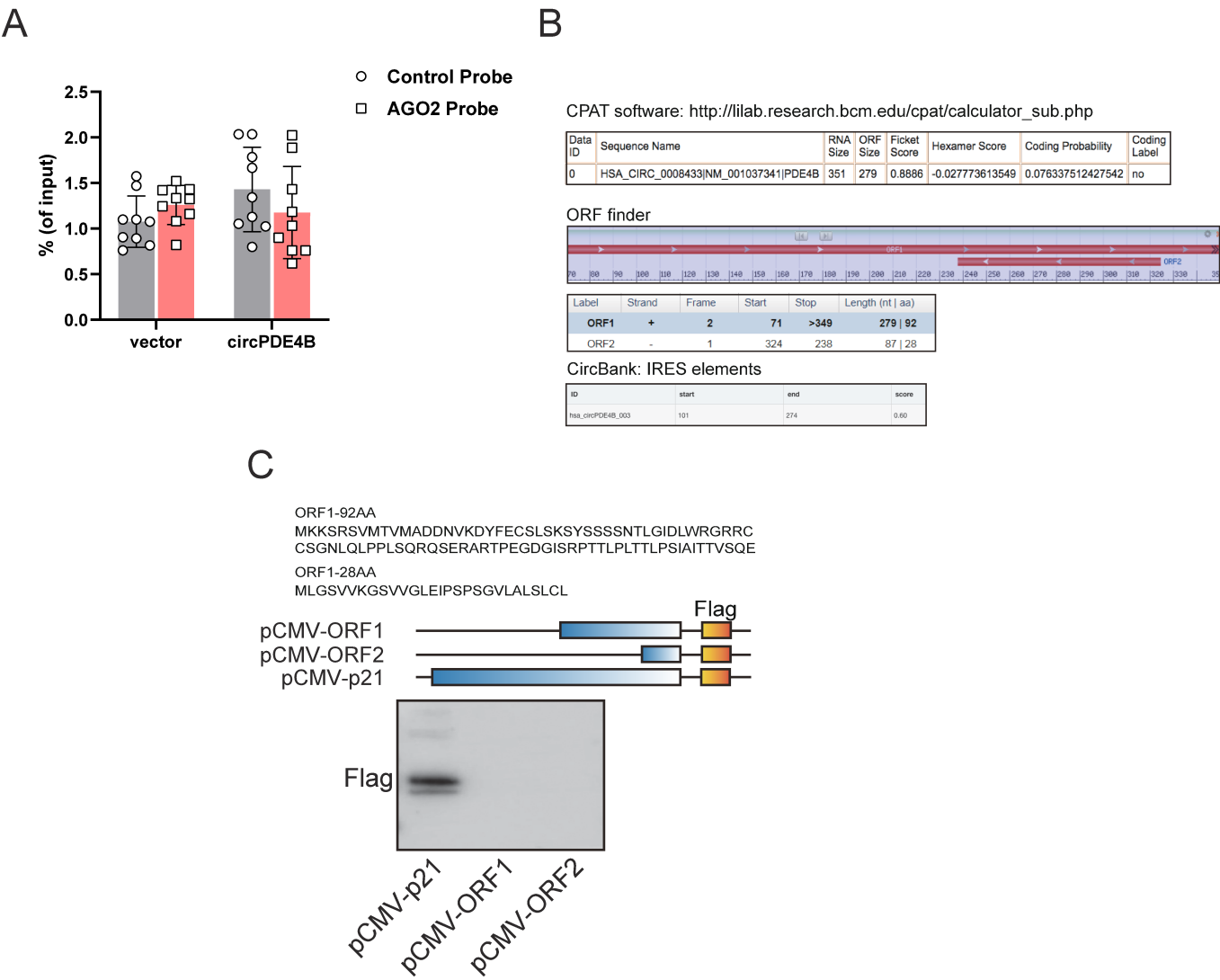

Supplement: Supplementary data [file annrheumdis-2021-219969supp005.pdf]

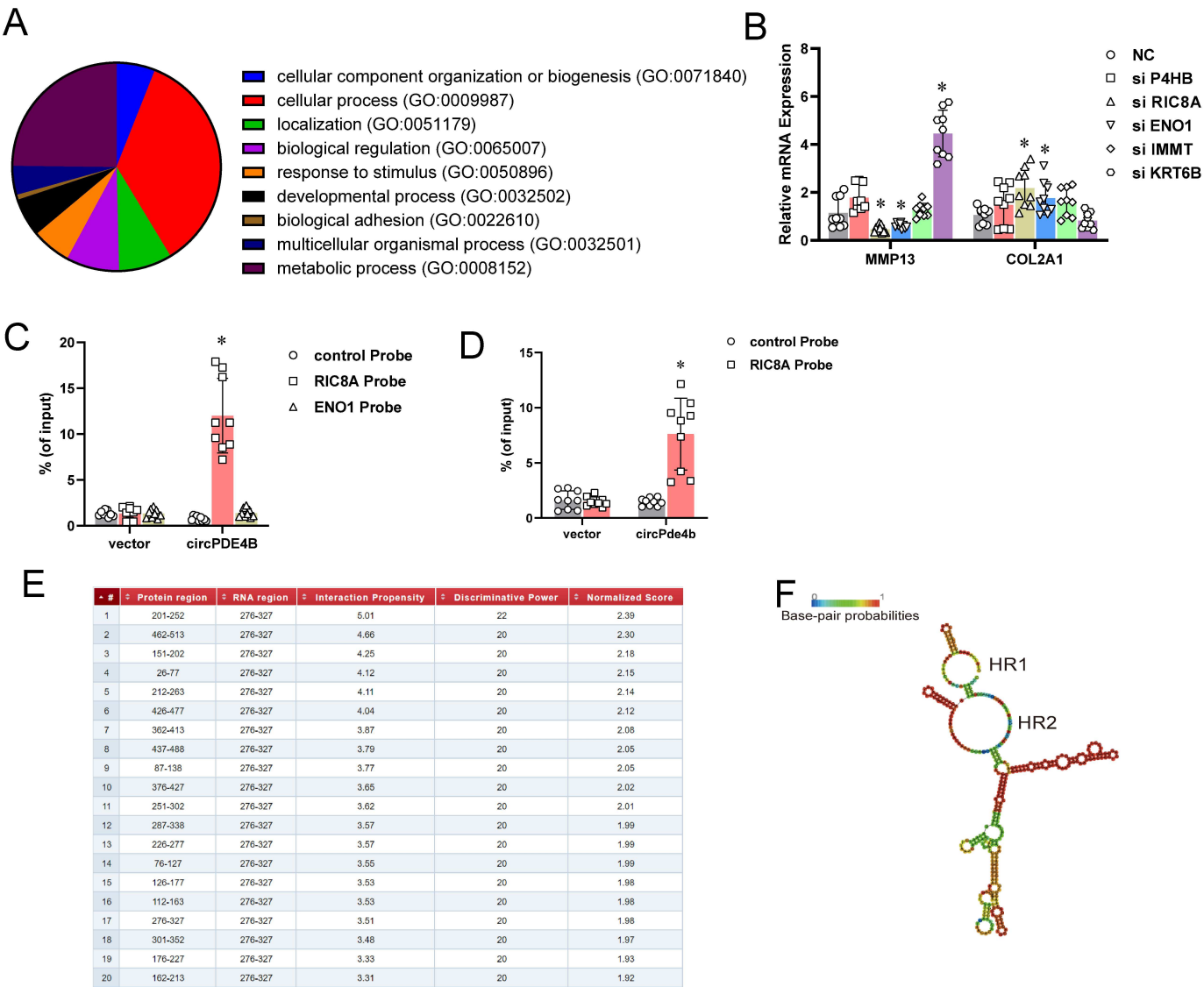

Supplement: Supplementary data [file annrheumdis-2021-219969supp006.pdf]

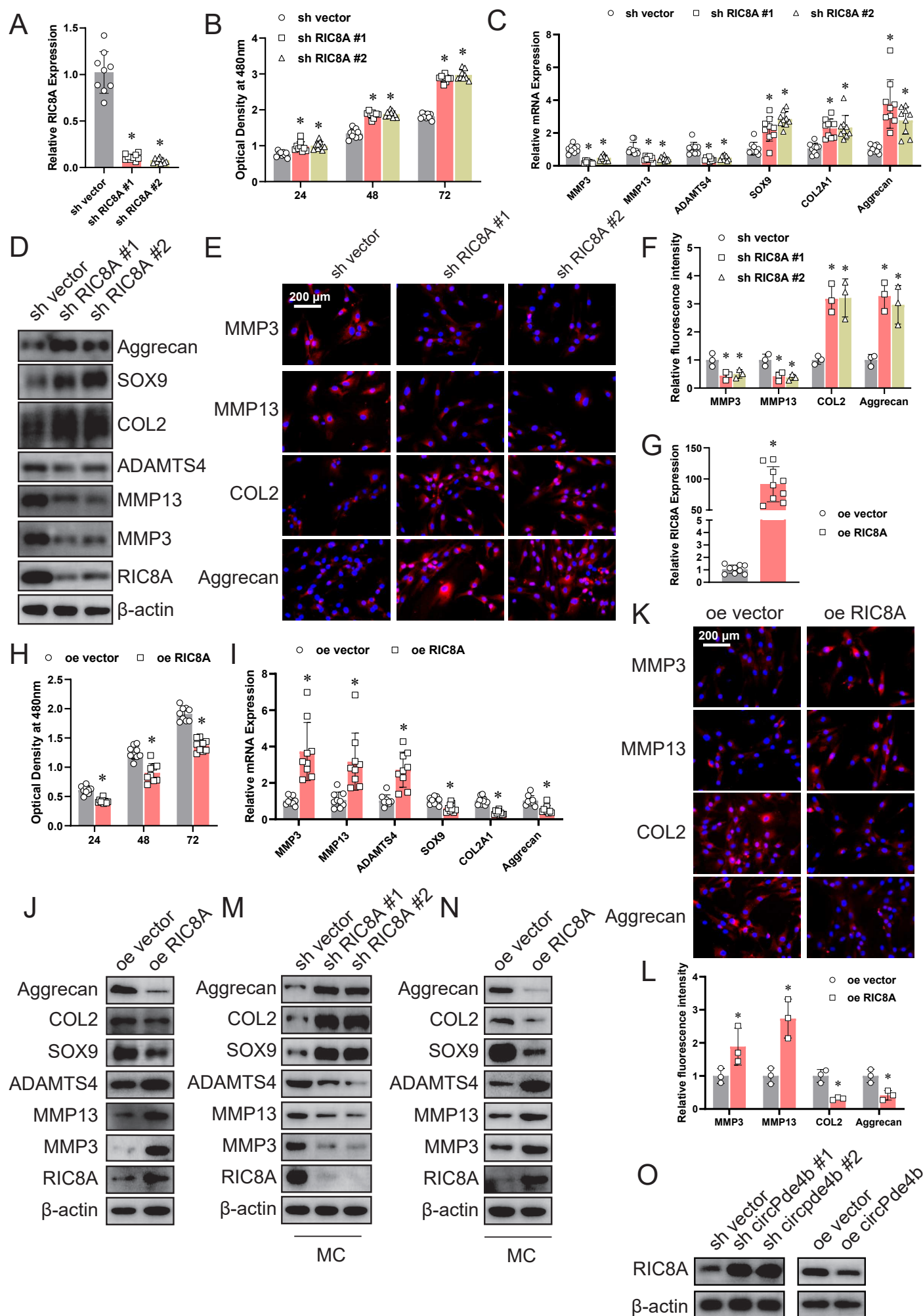

Supplement: Supplementary data [file annrheumdis-2021-219969supp007.pdf]

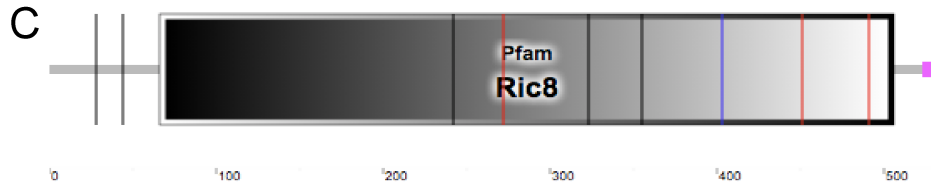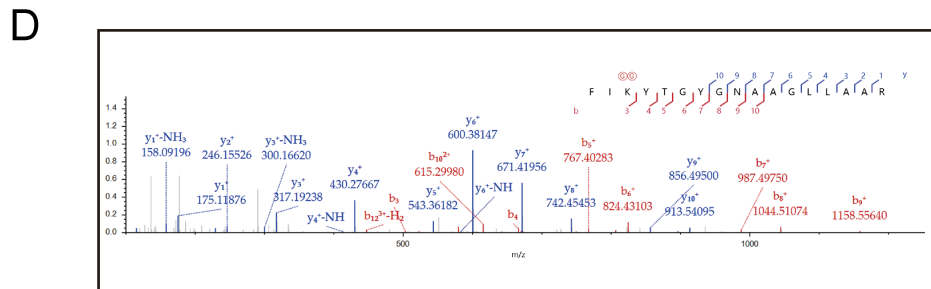

Supplement: Supplementary data [file annrheumdis-2021-219969supp008.pdf]

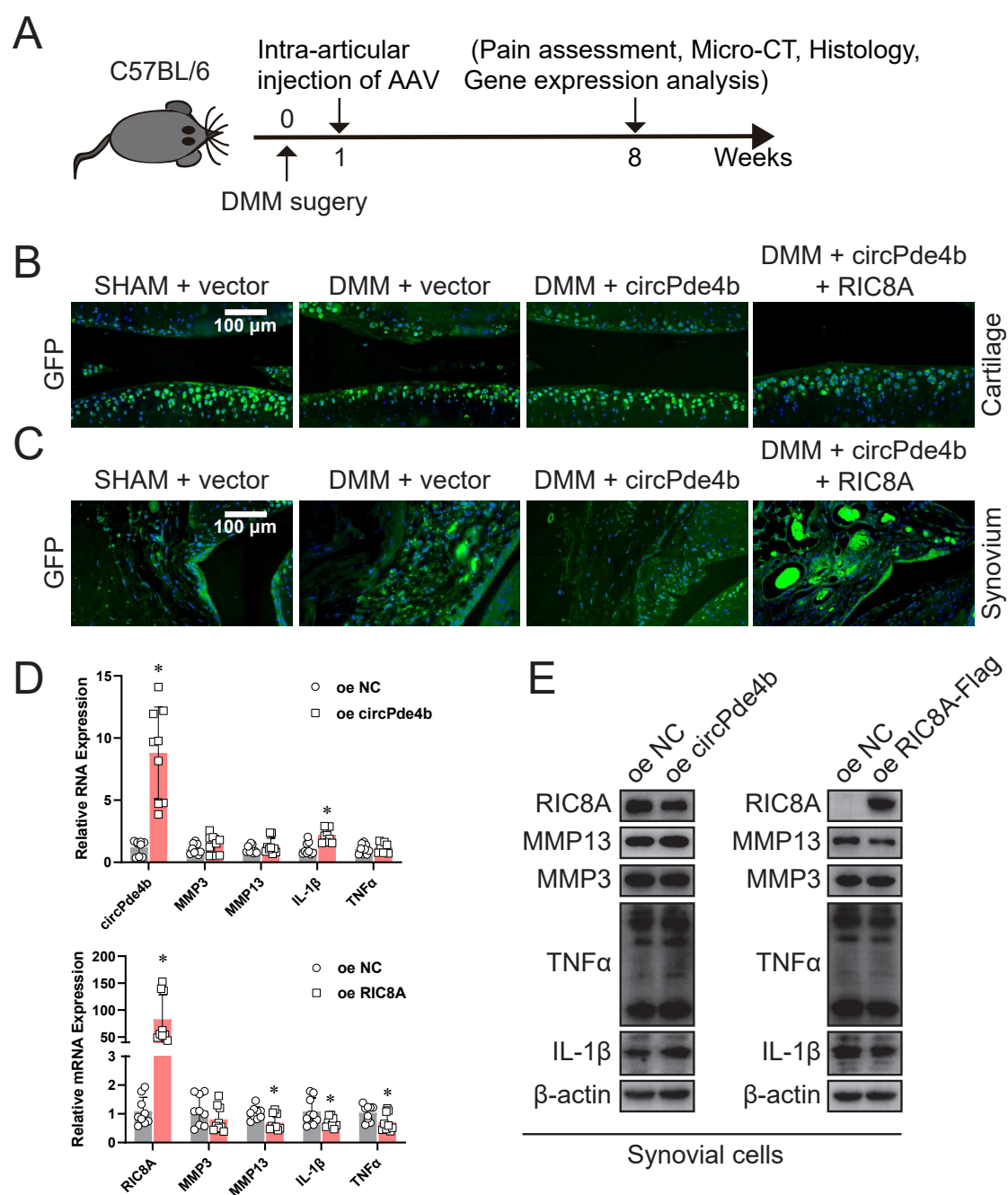

Supplement: Supplementary data [file annrheumdis-2021-219969supp009.pdf]

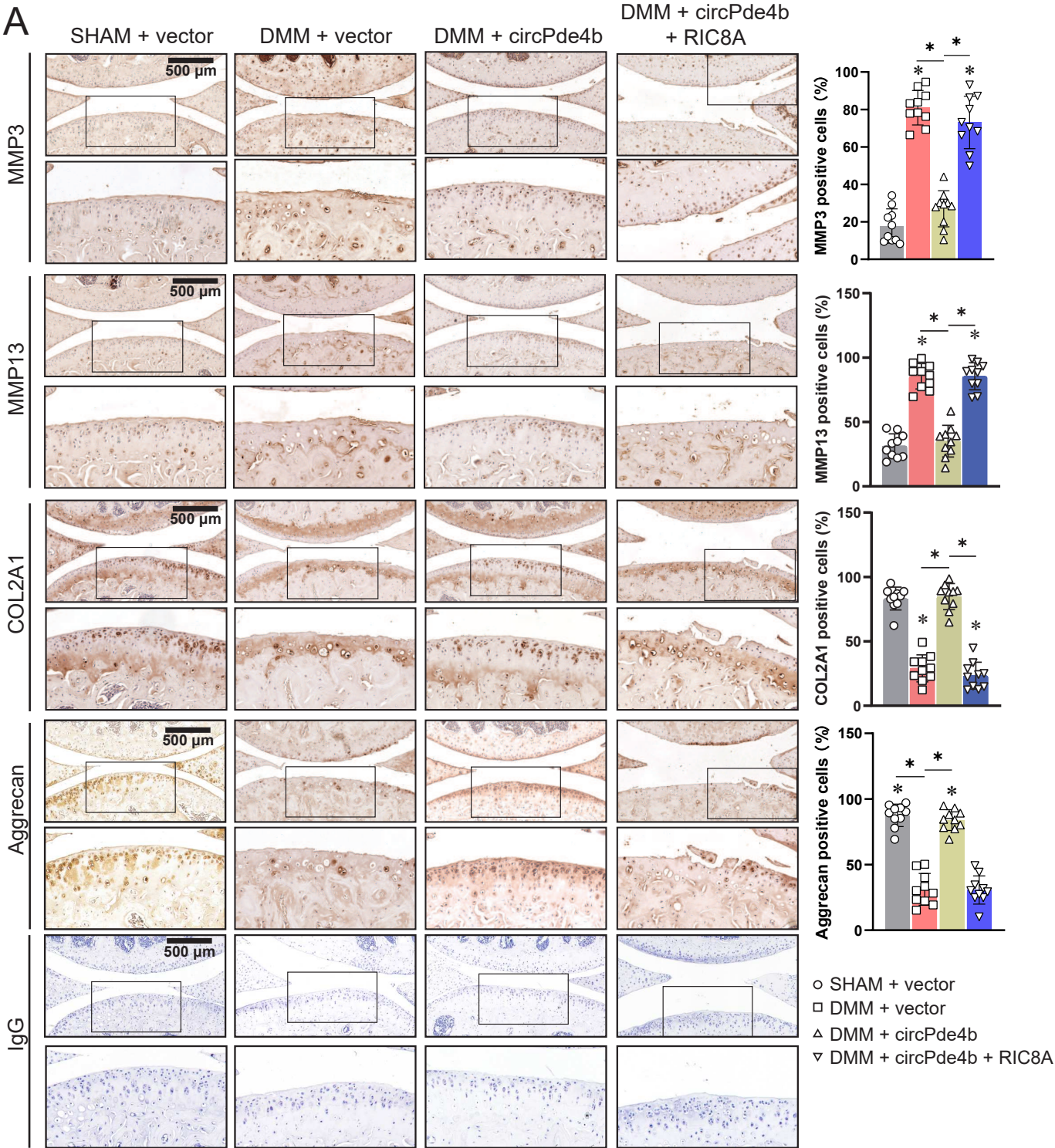

Supplement: Supplementary data [file annrheumdis-2021-219969supp010.pdf]
